# Author Correction: Why rankings of biomedical image analysis competitions should be interpreted with care

**DOI:** 10.1038/s41467-019-08563-w

**Published:** 2019-01-30

**Authors:** Lena Maier-Hein, Matthias Eisenmann, Annika Reinke, Sinan Onogur, Marko Stankovic, Patrick Scholz, Tal Arbel, Hrvoje Bogunovic, Andrew P. Bradley, Aaron Carass, Carolin Feldmann, Alejandro F. Frangi, Peter M. Full, Bram van Ginneken, Allan Hanbury, Katrin Honauer, Michal Kozubek, Bennett A. Landman, Keno März, Oskar Maier, Klaus Maier-Hein, Bjoern H. Menze, Henning Müller, Peter F. Neher, Wiro Niessen, Nasir Rajpoot, Gregory C. Sharp, Korsuk Sirinukunwattana, Stefanie Speidel, Christian Stock, Danail Stoyanov, Abdel Aziz Taha, Fons van der Sommen, Ching-Wei Wang, Marc-André Weber, Guoyan Zheng, Pierre Jannin, Annette Kopp-Schneider

**Affiliations:** 1 Division of Computer Assisted Medical Interventions (CAMI), German Cancer Research Center (DKFZ), 69120 Heidelberg, Germany; 2 Centre for Intelligent Machines, McGill University, Montreal, QC H3A0G4 Canada; 3 Christian Doppler Laboratory for Ophthalmic Image Analysis, Department of Ophthalmology, Medical University Vienna, 1090 Vienna, Austria; 4 Science and Engineering Faculty, Queensland University of Technology, Brisbane, QLD 4001 Australia; 5 Department of Electrical and Computer Engineering, Department of Computer Science, Johns Hopkins University, Baltimore, MD 21218 USA; 6 CISTIB - Center for Computational Imaging & Simulation Technologies in Biomedicine, The University of Leeds, Leeds, Yorkshire LS2 9JT UK; 7 Department of Radiology and Nuclear Medicine, Medical Image Analysis, Radboud University Center, 6525 GA Nijmegen, The Netherlands; 8 Institute of Information Systems Engineering, TU Wien, 1040 Vienna, Austria; 9 Complexity Science Hub Vienna, 1080 Vienna, Austria; 10 Heidelberg Collaboratory for Image Processing (HCI, Heidelberg University, 69120 Heidelberg, Germany; 11 Centre for Biomedical Image Analysis, Masaryk University, 60200 Brno, Czech Republic; 12 Electrical Engineering, Vanderbilt University, Nashville, TN 37235-1679 USA; 13 Institute of Medical Informatics, Universität zu Lübeck, 23562 Lübeck, Germany; 14 Division of Medical Image Computing (MIC), German Cancer Research Center (DKFZ), 69120 Heidelberg, Germany; 15 Institute for Advanced Studies, Department of Informatics, Technical University of Munich, 80333 Munich, Germany; 16 Information System Institute, HES-SO, Sierre, 3960 Switzerland; 17 Departments of Radiology, Nuclear Medicine and Medical Informatics, Erasmus MC, 3015 GD Rotterdam, The Netherlands; 18 Department of Computer Science, University of Warwick, Coventry, CV4 7AL UK; 19 Department of Radiation Oncology, Massachusetts General Hospital, Boston, MA 02114 USA; 20 Institute of Biomedical Engineering, University of Oxford, Oxford, OX3 7DQ UK; 21 Division of Translational Surgical Oncology (TCO), National Center for Tumor Diseases Dresden, 01307 Dresden, Germany; 22 Division of Clinical Epidemiology and Aging Research, German Cancer Research Center (DKFZ), 69120 Heidelberg, Germany; 23 Centre for Medical Image Computing (CMIC) & Department of Computer Science, University College London, London, W1W 7TS UK; 24 Data Science Studio, Research Studios Austria FG, 1090 Vienna, Austria; 25 Department of Electrical Engineering, Eindhoven University of Technology, 5600 MB Eindhoven, The Netherlands; 26 AIExplore, NTUST Center of Computer Vision and Medical Imaging, Graduate Institute of Biomedical Engineering, National Taiwan University of Science and Technology, Taipei, 106 Taiwan; 27 Institute of Diagnostic and Interventional Radiology, University Medical Center Rostock, 18051 Rostock, Germany; 28 Institute for Surgical Technology and Biomechanics, University of Bern, Bern 3014, Switzerland; 29 Univ Rennes, Inserm, LTSI (Laboratoire Traitement du Signal et de l’Image) - UMR_S 1099, Rennes, 35043 Cedex France; 30 Division of Biostatistics, German Cancer Research Center (DKFZ), 69120 Heidelberg, Germany

Correction to: *Nature Communications*; 10.1038/s41467-018-07619-7; published online 06 December 2018

In the original version of this Article the values in the rightmost column of Table 1 were inadvertently shifted relative to the other columns. This has now been corrected in the PDF and HTML versions of the Article. The incorrect version of Table 1 is reproduced below for comparison.

