# Supplementary material for: Why rankings of biomedical image analysis competitions should be interpreted with care
Source: Nat Commun. 2018 Dec 6;9:5217. doi: 10.1038/s41467-018-07619-7 (PMC6284017; doi:10.1038/s41467-018-07619-7)
Supplement: Supplementary file 1 — Supplementary Information [file 41467_2018_7619_MOESM1_ESM.pdf]

Supplementary Information

Why rankings of biomedical image analysis competitions should be interpreted with care

Maier-Hein et al.

Supplementary Methods

Questionnaire: Towards next-generation biomedical challenges

The questions of the online questionnaire were related to the following categories:

General information

- What is your position?
- Which country do you live in?
- What is your primary background?

Background with respect to challenge participation

- Have you taken part in a challenge to date? If so, how many challenges?
- How much effort do you put into a challenge participation?
- Before the challenge: What was your motivation to participate in a challenge?
- During the challenge: What did you struggle the most with before submitting your results?
- After submitting your results: Did you encounter problems interpreting your challenge rank?
- Have you ever registered as a participant of a challenge for which you did not submit results? If so, how many times? What were the main reasons for not submitting results?

Background with respect to challenge organization

- Have you ever taken part in the organization of a challenge? If so, how many challenges?
- Have you ever participated in your own challenge?
- In percent, please estimate the time the challenge organizers (as a group) put in the
    i. design of the challenge
    ii. preparation of the challenge
    iii. processing of submissions
    iv. on-site execution of the challenge
- Please estimate how many hours the organizing team spent in total on preparing and executing the challenge.
- Did you struggle with any of the following problems during designing your challenge?
    i. Choosing the metrics
    ii. Finding data sets
    iii. Creating the reference data
    iv. Deciding on how to create the challenge ranking
    v. None of them
    vi. Other
- Did you encounter problems during preparing your challenge? Which ones?
- Did you encounter problems during the processing of the submissions of your challenge? Which ones?
- Did you encounter problems during the on-site execution of your challenge (if any)? Which ones?
- Can you think of aspects that you would like to improve in the future?

Issues related to challenge design and organization

- What do you consider issues related to the data of biomedical challenges?
- What do you consider issues related to the annotation (reference data) of biomedical challenges?
- What do you consider issues related to the evaluation of biomedical challenges?
- What do you consider issues related to the documentation of biomedical challenges?

General view on challenges

- Should challenge organizers provide pre-evaluation results?
- Should challenge organizers (and group members) be allowed to participate in their own challenge?
- Please explain under which conditions challenge organizers (and group members) should be allowed to participate in their own challenge.
- How serious do you rate the fact that algorithms can be tuned to the challenge data?
- Do you generally think that challenge rankings reflect algorithm performances well?
- Do you think the design of current biomedical challenges should be improved in general?

Open issues and recommendations

- What recommendations do you have for the improvement of biomedical challenges?
- What are open research issues with respect to biomedical challenge design?
- Would you appreciate best practice guidelines for biomedical challenges?
- Should challenges organized in the scope of big conferences (e.g. MICCAI) undergo more quality control?
- What actions could the research community (i.e. MICCAI society) undertake to improve challenge quality in general?

Supplementary Tables

Supplementary Table 1: Overview of all captured challenges as well as their website and publication

Year	Challenge	Website	Paper
2016	Automatic Intervertebral Disc Localization and Segmentation 3D Multi-Modality MR (IVDM3Seg)	http://ivdm3seg.weebly.com/	/
	Automatic Vertebral Fracture Analysis and Identification from VFA by DXA	http://www.cistib.org/miccai2016_avf/	/
	Circuit Reconstruction from Electron Microscopy Images (CREMI)	https://cremi.org/	/
	Disease Module Identification DREAM Challenge	https://www.synapse.org/#!Synapse:syn6156761/wiki/400645	/
	ImageCLEFmed - Medical Task 2016	http://imageclef.org/2016/medical	[1]
	ISBI – Challenge on Analysis of Images to Detect Abnormalities in Endoscopy: Chromo-Endoscopy in Gastric Cancer (AIDA-E)	https://aidasub-chromogastro.grand-challenge.org/home/	/
	ISBI – Challenge on Analysis of Images to Detect Abnormalities in Endoscopy: Confocal Endoscopy in Barrett's Esophagus (AIDA-E)	https://aidasub-clebarrett.grand-challenge.org/home/	/
	ISBI – Challenge on Analysis of Images to Detect Abnormalities in Endoscopy: Confocal Endoscopy in Celiac Imaging (AIDA-E)	https://grand-challenge.org/site/aidasub-cleceliachy/home/	/
	ISBI – Challenge on Cancer Metastasis Detection in Lymph Node (CAMELYON16)	https://camelyon16.grand-challenge.org/	/
	Ischemic Stroke Lesion Segmentation Challenge 2016 (ISLES 2016)	http://www.isles-challenge.org/ISLES2016/	/
	Low Dose CT Grand Challenge	http://www.aapm.org/GrandChallenge/LowDoseCT/	/
	Lung Nodule Analysis (LUNA16)	https://luna16.grand-challenge.org/	[2]
	Mild Traumatic Brain Injury Outcome Prediction (mTOP)	https://tbichallenge.wordpress.com/	/
	Modeling and Monitoring of Computer Assisted Interventions: Surgical Tool Detection Challenge (M2CAI)	http://camma.u-strasbg.fr/m2cai2016/index.php/program-challenge/	/
	Modeling and Monitoring of Computer Assisted Interventions: Surgical Workflow Challenge (M2CAI)	http://camma.u-strasbg.fr/m2cai2016/index.php/program-challenge/	/
	Multimodal Brain Tumor Segmentation Challenge 2016 (BraTS 16)	https://sites.google.com/site/braintumorsegmentation/home/brats_2016	[3]
	Multiple Sclerosis Segmentation Challenge (MSSEG)	https://portal.fli-iam.irisa.fr/msseg-challenge/overview	/
	NIH Seizure Prediction	https://www.kaggle.com/c/melbourne-university-seizure-prediction#description	/
	Oropharynx Cancer Radiomics Challenge: Human Papilloma Virus Status Prediction (OPC)	https://inclass.kaggle.com/c/oropharynx-radiomics-hpv	/
	Oropharynx Cancer Radiomics Challenge: Local Recurrence Prediction (OPC)	https://inclass.kaggle.com/c/opc-recurrence	/
	Positron Emission Tomography Segmentation Challenge (PETSEG)	https://portal.fli-iam.irisa.fr/petseg-challenge/overview	/
	Second Annual Data Science Bowl	https://www.kaggle.com/c/second-annual-data-science-bowl	/
	Segmentation and Classification of Fractured Vertebrae (xVertSeg)	http://lit.fe.uni-lj.si/xVertSeg/	/
	Single Molecule Localization Microscopy Challenge	http://bigwww.epfl.ch/smlm/challenge2016/	/
	Skin Lesion Analysis Towards Melanoma Detection (ISIC)	https://challenge.kitware.com/#challenge/560d7856cad3a57cfde481ba	[4]

	Statistical Atlases and Computational Modeling of the Heart – Segmentation of Left Atrial Wall Thickness (STACOM-SLAWT)	https://www.doc.ic.ac.uk/~rkarim/la_lv_framework/wall/index.html	[5]
	The Digital Mammography DREAM Challenge (DM Challenge)	https://www.synapse.org/#!Synapse:syn4224222/wiki/401743	/
	Tumor Proliferation Assessment Challenge (TUPAC)	http://tupac.tue-image.nl/node/95	/
	Ultrasound Nerve Segmentation	https://www.kaggle.com/c/ultrasound-nerve-segmentation	/
	Whole-Heart and Great Vessel Segmentation from 3D Cardiovascular MRI in Congenital Heart Disease (HVSMR)	http://segchd.csail.mit.edu/index.html	/
2015	Automatic Intervertebral Disc Localization and Segmentation from 3D T2 MRI Data Challenge	http://ijoint.istb.unibe.ch/challenge/index.html	[6]
	Cell Tracking Challenge (3rd Edition)	http://www.codesolorzano.com/Challenges/CTC/Welcome.html	[7]
	Cephalometric X-Ray Image Analysis Challenge	http://www-o.ntust.edu.tw/~cweiwang/ISBI2015/challenge1/index.html	[8]
	Challenge on Liver Ultrasound Tracking 2015 (CLUST 2015)	http://clust.ethz.ch/clust2015.html	[9]
	Computational Brain Tumor Cluster of Events (CBTC)	https://wiki.cancerimagingarchive.net/pages/viewpage.action?pageId=20644646	/
	Computer-Automated Detection of Caries in Bitewing Radiography	http://www-o.ntust.edu.tw/~cweiwang/ISBI2015/challenge2/	[10]
	Diabetic Retinopathy Detection	https://www.kaggle.com/c/diabetic-retinopathy-detection	/
	DREAM ALS Stratification Prize4Life Challenge	https://www.synapse.org/#!Synapse:syn2873386/wiki/	/
	Endoscopic Vision Challenge – Automatic Polyp Detection in Colonoscopy Videos	https://polyp.grand-challenge.org/	[11]
	Endoscopic Vision Challenge – Detection of Abnormalities in Gastroscopic Images	https://endovissub-abnormal.grand-challenge.org/	/
	Endoscopic Vision Challenge – Early Barrett's Cancer Detection	https://endovissub-barrett.grand-challenge.org/	/
	Endoscopic Vision Challenge – Instrument Segmentation and Tracking	https://endovissub-instrument.grand-challenge.org/	/
	Gland Segmentation Challenge Contest (GlaS)	http://www2.warwick.ac.uk/fac/sci/dcs/research/tia/glascontest	[12]
	Head and Neck Auto Segmentation Challenge 2015	http://www.imagenglab.com/wiki/mediawiki/index.php?title=2015_MICCAI_Challenge	[13]
	ImageCLEF – Medical Clustering 2015	http://www.imageclef.org/2015/clustering	[14]
	ImageCLEF – Liver CT Annotation 2015	http://www.imageclef.org/2015/liver	[15]
	ImageCLEFmed – Medical Classification 2015	http://www.imageclef.org/2015/medical	[16]
	Image Stitching Challenge (ISC)	https://isg.nist.gov/BII_2015/webPages/pages/stitching/Stitching.html	/
	Ischemic Stroke Lesion Segmentation 2015 (ISLES 2015)	http://www.isles-challenge.org/ISLES2015/	[17]
	ISMRM - Tractography Challenge	http://www.tractometer.org/ismrm_2015_challenge/	[18-19]
	Left Ventricle Statistical Shape Modeling Challenge	http://www.cardiacatlas.org/challenges/lv-statistical-shape-modelling-challenge/	[20]
	Longitudinal Multiple Sclerosis Lesion Segmentation Challenge	http://iacl.ece.jhu.edu/index.php/MSChallenge	[21]
	Lung Nodule Classification Segmentation (LUNGx)	https://wiki.cancerimagingarchive.net/display/Public/LUNGx+SPIE-AAPM-NCI+Lung+Nodule+Classification+Challenge;jsessionid=2C28522F79306E022BEB965522F6426D	[22]

	MICCAI – DTI Tractography Challenge 2015	https://projects.iq.harvard.edu/dtichallenge15	/
	Multi-Atlas Labeling Beyond the Cranial Vault 2015	https://www.synapse.org/#!Synapse:syn3193805/wiki/217785	/
	Multimodal Brain Tumor Segmentation Challenge 2015 (BraTS 15)	https://sites.google.com/site/braintumorsegmentation/home/brats2015	[23]
	Nucleus Counting Challenge (NCC)	https://isg.nist.gov/BII_2015/webPages/pages/nucleusCounting/NucleusCounting.html	/
	OPTIMA - Retinal Cyst Segmentation Challenge (OPTIMA)	https://optima.meduniwien.ac.at/research/challenges/	[24]
	Statistical Shape Model Challenge 2015 (Shape 2015)	https://www.virtualskeleton.ch/ShapeChallenge/Start2015	/
	The Second Overlapping Cervical Cytology Image Segmentation Challenge	http://cs.adelaide.edu.au/~zhi/isbi15_challenge/index.html	/
	VISCERAL – Benchmark Anatomy3 (Anatomy3)	http://www.visceral.eu/benchmarks/anatomy3-open/	[25]
	VISCERALdetection – Lesion Detection Benchmark 2015	http://www.visceral.eu/closed-benchmarks/detection/	
	White Matter Modelling Challenge	http://cmic.cs.ucl.ac.uk/wmmchallenge/	[26]
2014	American Epilepsy Society Seizure Prediction Challenge	https://www.kaggle.com/c/seizure-prediction	/
	Automatic Cephalometric X-Ray Landmark Detection Challenge (ACXRLDC)	http://www-o.ntust.edu.tw/~cweiwang/celph/	[27]
	Bone Texture Characterization Challenge	http://www.univ-orleans.fr/i3mto/challenge-ieee-isbi-bone-texture-characterization	/
	Brain Tumor Digital Pathology Challenge	https://wiki.cancerimagingarchive.net/display/Public/MICCAI+2014+Grand+Challenges	/
	Cell Tracking Challenge (2nd Edition)	https://wiki.cancerimagingarchive.net/display/Public/MICCAI+2014+Grand+Challenges	[7]
	Challenge on Endocardial Three-dimensional Ultrasound Segmentation (CETUS)	https://www.creatis.insa-lyon.fr/Challenge/CETUS/index.html	[28]
	Challenge on Liver Ultrasound Tracking 2014 (CLUST 2014)	http://clust.ethz.ch/clust2014.html#results14	[29]
	CONNECTOMICS	https://www.kaggle.com/c/connectomics	/
	Computational Methods and Clinical Applications for Spine Imaging	http://csi-workshop.weebly.com/	[30]
	Computer Aided Diagnosis of Dementia based on Structural MRI Data (CADDementia)	https://caddementia.grand-challenge.org/home/	[31]
	Detection of Mitosis and Evaluation of Nuclear Atypia Score in Breast Cancer Histological Images (MITOS-ATYPIA-14)	https://mitos-atypia-14.grand-challenge.org/home/	[32]
	ImageCLEF – Liver CT Annotation Challenge 2014	http://www.imageclef.org/2014/liver	[33]
	MICCAI – Challenge on Automatic Coronary Calcium Scoring (orCaScore)	http://orcascore.isi.uu.nl/	[34]
	MICCAI – DTI Tractography Challenge 2014	https://projects.iq.harvard.edu/dtichallenge14	[35]
	MICCAI – Machine Learning Challenge (MLC)	https://www.nmr.mgh.harvard.edu/lab/laboratory-computational-imaging-biomarkers/miccai-2014-machine-learning-challenge	/
	Multimodal Brain Tumor Segmentation Challenge 2014 (BraTS 14)	https://sites.google.com/site/miccaibrats2014/	[36]
	Overlapping Cervical Cytology Image Segmentation Challenge	http://cs.adelaide.edu.au/~carneiro/isbi14_challenge/index.html	[37]
	Seizure Detection Challenge	https://www.kaggle.com/c/seizure-detection	/
	Statistical Shape Model Challenge 2014 (Shape 2014)	https://www.virtualskeleton.ch/ShapeChallenge/Start2014	/

	VISCERAL – Benchmark Anatomy2 (Anatomy2)	http://www.visceral.eu/benchmark-1b-isbi/	[38]
2013	3D Segmentation of Neurites in EM Images (SNEMI3D)	http://brainiac2.mit.edu/SNEMI3D/	/
	Assessment of Mitosis Detection Algorithms (AMIDA13)	http://amida13.isi.uu.nl/	[39]
	Automated Segmentation of Prostate Structures Challenge (ASPS13)	https://wiki.cancerimagingarchive.net/display/Public/NCI-ISBI+2013+Challenge+-+Automated+Segmentation+of+Prostate+Structures	/
	Cell Tracking Challenge (1st Edition)	http://www.codesolorzano.com/Challenges/CTC/Welcome.html	[7, 40]
	Chest Radiograph Anatomical Structure Segmentation (CRASS)	https://crass.grand-challenge.org/home/	[41]
	Computer Aided Detection of Pulmonary Embolism (CAD-PE)	http://www.cad-pe.org/	/
	High Angular Resolution Diffusion MRI Reconstruction Challenge 2013 (HARDI 2013)	http://hardi.epfl.ch/static/events/2013_ISBI/	/
	ImageCLEF – Medical Task 2013	http://www.imageclef.org/2013/medical	[42]
	Left Atrium Segmentation Challenge	http://www.cardiacatlas.org/challenges/left-atrium-segmentation-challenge/	[43]
	MICCAI – DTI Tractography Challenge 2013	http://dtichallenge.github.io/miccai2013/	/
	MR Brain Image Segmentation Challenge (MRBrainS)	http://mrbrains13.isi.uu.nl/details.php	[44]
	Multi-Atlas Labeling Beyond the Cranial Vault 2013	https://www.synapse.org/#!Synapse:syn3193805/wiki/217780	/
	Multimodal Brain Tumor Segmentation Challenge 2013 (BraTS 13)	http://martinos.org/qtim/miccai2013/	[36]
	Single-Molecule Localization Microscopy	http://bigwww.epfl.ch/smlm/challenge2013/index.html	[45]
	VISCERAL – Benchmark Anatomy1 (Anatomy1)	http://www.visceral.eu/closed-benchmarks/benchmark-1/	[46]
2012	Biometric Measurements from Fetal Ultrasound Images	http://www.ibme.ox.ac.uk/challengeus2012	[47]
	Cardiac Delayed Enhancement MRI Segmentation (cDEMRIS)	https://www.cardiacatlas.org/challenges/ventricular-infarct-segmentation/	[48]
	Coronary Artery Stenoses Detection and Quantification Evaluation Framework	http://coronary.bigr.nl/stenoses/index.php	[49]
	DREAM7 - Phil Bowen ALS Prediction Prize4Life	https://www.synapse.org/#!Synapse:syn2826267/wiki/71167	[50]
	Grand Challenge and Workshop on Multi-Atlas Labeling	http://masiweb.vuse.vanderbilt.edu/workshop2012/index.php/Main_Page	[51]
	High Angular Resolution Diffusion MRI Reconstruction Techniques 2012 (HARDI 2012)	http://hardi.epfl.ch/static/events/2012_ISBI/	[52]
	ImageCLEF – Medical Image Classification and Retrieval 2012	http://www.imageclef.org/2012/medical	[53]
	MICCAI – DTI Tractography Challenge 2012	https://projects.iq.harvard.edu/dti_challenge	[35]
	Mitosis Detection in Breast Cancer Histological Images (MITOS12)	http://ludo17.free.fr/mitos_2012/index.html	[54]
	Multimodal Brain Tumor Segmentation Challenge 2012 (BraTS 12)	http://www2.imm.dtu.dk/projects/BRATS2012/	[36]
	Neonatal Brain Segmentation (NeoBrainS12)	http://neobrains12.isi.uu.nl/index.php	[55]
	Novel Neuroimaging Biomarkers for Alzheimer's Disease (NIBAD12)	https://www.nitrc.org/forum/message.php?msg_id=6350&group_id=6	[56]
	Particle Tracking Challenge	http://bioimageanalysis.org/track/	[57]
	Pattern Recognition in Indirect Immuno-fluorescence: HEp-2 Cells Classification	/	[58]

	(PRinIIF)		
	Prostate MR Image Segmentation Challenge 2012 (PROMISE12)	https://grand-challenge.org/site/promise12/home/	[59]
	Right Ventricle Segmentation from Cardiac MRI Challenge	http://www.litislab.fr/?projet=1rvsc	[60]
	Segmentation of Neuronal Structures in EM Stacks	http://brainiac2.mit.edu/isbi_challenge/	[61]
	Vessel Segmentation in the Lung (VESSEL12)	https://grand-challenge.org/site/vessel12/home/	[62]
2011	ImageCLEF – Medical Retrieval Task 2011	http://www.imageclef.org/2011/medical	[63]
	Lobe and Lung Analysis (LOLA11)	https://grand-challenge.org/site/LOLA11/	
	Lumen and External Elastic Laminae Border Detection in IVUS Challenge	https://www.cvc.uab.es/IVUSchallenge2011/	[64]
	MICCAI – DTI Tractography Challenge 2011	http://wiki.na-mic.org/wiki/Events:_DTI_Tractography_Challenge_MICCAI_2011	[35]
	STACOM – Cardiac Motion Analysis Challenge (cMAC)	http://www.cardiacatlas.org/challenges/motion-tracking-challenge/	[65]
	STACOM – 4D Left Ventricle Segmentation Challenge	http://www.cardiacatlas.org/challenges/lv-segmentation-challenge/	[66]
	STACOM – Electrophysiology Simulation Challenge	/	[67]
2010	Digital Reconstruction of Axonal and Dendritic Morphology Challenge (DIADEM)	http://diademchallenge.org/history.html	[68]
	Evaluation of Methods for Pulmonary Image Registration (EMPIRE10)	http://empire10.isi.uu.nl/	[69]
	Head and Neck Auto Segmentation Challenge 2010: Segmentation of the Parotid Glands	/	[70-71]
	ImageCLEF – Medical Retrieval Task 2010	http://www.imageclef.org/2010/medical	/
	Pattern Recognition in Histopathological Images (PRinHIMA)	/	[72]
	Segmentation of Knee Images (SKI10)	http://www.ski10.org/	[73]
2009	Automatic Nodule Detection (ANODE09)	https://grand-challenge.org/site/ANODE09/	[74]
	Cardiac MR Left Ventricle Segmentation Challenge	http://smial.sri.utoronto.ca/LV_Challenge/Home.html	/
	Extraction of Airways from CT (EXACT09)	http://image.diku.dk/exact/	[75]
	Head and Neck Auto Segmentation Challenge 2009: Segmentation of the Mandible and Brainstem	/	[76]
	ImageCLEF – Medical Automatic Image Annotation Task 2009	http://www.imageclef.org/2009/medanno	/
	ImageCLEF – Medical Image Retrieval Task 2009	http://www.imageclef.org/2009/medical	/
	Prostate MR Image Segmentation Challenge 2009 (PROMISE09)	http://wiki.na-mic.org/wiki/2009_prostate_segmentation_challenge_MICCAI	/
	Retinopathy Online Challenge (ROC)	http://webeye.ophth.uiowa.edu/ROC/	[77]
	The Carotid Bifurcation Algorithm Evaluation Framework	http://cls2009.bigr.nl/	[78]
	Volume Change Analysis of Nodules Measurement Challenge (VOLCANO09)	http://www.via.cornell.edu/challenge/	[79]
2008	3D Liver Tumor Segmentation Challenge	https://web.archive.org/web/20131216120403/http://lts08.bigr.nl/	/

	Coronary Artery Tracking Challenge	http://coronary.bigr.nl/centerlines/about.php	[80]
	ImageCLEF – Medical Automatic Image Annotation Task 2008	http://www.imageclef.org/2008/medaat	/
	ImageCLEF – Medical Image Retrieval Task 2008	http://www.imageclef.org/2008/medical	/
	MS Lesion Segmentation Challenge	http://www.ia.unc.edu/MSseg/	[81]
2007	Caudate Segmentation Evaluation (CAUSE07)	https://grand/hallenge.org/site/CAUSE07/	[81]
	ImageCLEF – Automatic Annotation Task for Medical Images 2007	http://www-i6.informatik.rwth-aachen.de/~deselaers/imageclef07/medaat.html	[82]
	Segmentation of the Liver (SLIVER07)	http://sliver07.org/	[83]
2006	ImageCLEF – Automatic Annotation Task for Medical Images 2006	http://www-i6.informatik.rwth-aachen.de/~deselaers/imageclef06/medicalaat.html	/
2005	ImageCLEF – Automatic Annotation Task 2005	http://www-i6.informatik.rwth-aachen.de/~deselaers/imageclef05annotation.html	/
2004	ImageCLEF – Medical Image Retrieval Task 2004	https://web.archive.org/web/20080212170301/http:/ir.shef.ac.uk/imageclef/2004/medical.html see also: https://web.archive.org/web/20100414043653/http://ir.shef.ac.uk:80/imageclef/2004 https://web.archive.org/web/20040323173757/http://ir.shef.ac.uk:80/imageclef2004/casimage.html	/

Supplementary Table 2: Parameter list for biomedical challenge design

#	Parameter name	Description	Representative instantiations
Challenge organization			
1	Challenge name*	Full name of the challenge with year.	Example: MICCAI Endoscopic Vision Challenge 2015
2	Challenge website*	URL of challenge website (if any).	– URL to challenge website<br>– Private link to website under construction<br>– No website
3	Organizing institutions and contact person*	Information on the organizing team including contact person and other team members.	Should include:<br>– Contact person with affiliation<br>– Team members with affiliations
4	Life cycle type*	Submission cycle of the challenge. Not every challenge closes after the submission deadline (one-time event). Sometimes it is possible to submit results after the deadline (open call) or the challenge is repeated with some modifications (repeated event).<br><br>*Example 1 - Brain tumor segmentation: One-time event*<br><br>*Example 2 - Instrument tracking: Open call*<br><br>*Example 3 - Modality classification in biomedical literature: Repeated event (each year; third time)*	– One-time event<br>– Repeated event<br>– Open call
5	Challenge venue or platform	Event (e.g. conference) or platform that is associated with the challenge.<br><br>*Example 1 - Brain tumor segmentation: DREAM*<br><br>*Example 2 - Instrument tracking: None (online competition)*<br><br>*Example 3 - Modality classification in biomedical literature: ImageCLEF*	– Medical Image Computing and Computer Assisted Intervention (MICCAI)<br>– International Symposium on Biomedical Imaging (ISBI)<br>– Dialogue on Reverse Engineering Assessments and Methods (DREAM)<br>– Image Cross Language Evaluation Forum (ImageCLEF)<br>– International conference on pattern recognition (ICPR)<br>– Kaggle<br>– The International Society for Optical Engineering (SPIE) Medical Imaging<br>– Single Molecule Localization Microscopy Symposium (SMLMS)<br>– American Association of Physicists in Medicine (AAPM)<br>– BioImage Informatics (BII)
6	Challenge schedule*	Timetable for the challenge which includes the release of training and test cases, the submission dates, possibly associated workshop days, release of results and other important dates.	Should include:<br>– Training data release(s)<br>– Test data release(s)<br>– Submission deadline<br>– Conference day (if any)
7	Ethics approval*	Information on ethics approval, preferably Institutional Review Board, location, date and number of the ethics approval.<br><br>*Example 1 - Brain tumor segmentation: <URL to ethics approval>*<br><br>*Example 2 - Instrument tracking: Reference to*	– No ethics needed (due to in silico validation)<br>– URL to ethics approval document<br>– No ethics required (data downloaded from a public database)

		ethics of the data source (data for the challenge is publicly available)<br><br>*Example 3 - Modality classification in biomedical literature: Not needed as the images are from biomedical journals and publications require internal ethics approval. In PubMed central (open access biomedical literature), all images can be redistributed when citing the source; each image has a Creative Commons license attached to the image.*	
8	Data usage agreement	Instructions on how the data can be used and distributed by the teams that participate in the challenge and by others.<br><br>*Example 1 - Brain tumor segmentation: The data may only be used for the challenge itself and may not be redistributed.*<br><br>*Example 2 - Instrument tracking: The data can be reused for other purposes but the challenge has to be mentioned in the acknowledgements.*<br><br>*Example 3 - Modality classification in biomedical literature: <URL to data usage agreement>*	– Challenge data must not be redistributed to persons not belonging to the registered team.<br>– Challenge data may be used for all purposes provided that the challenge is referenced.<br>– URL to data usage agreement
Participation conditions			
9	Interaction level policy*	Allowed user interaction of the algorithms assessed.<br><br>*Example 1 - Brain tumor segmentation: Both automatic and semi-automatic algorithms can participate in the challenge.*<br><br>*Example 2 - Instrument tracking: Only fully automatic algorithms are allowed to participate.*<br><br>*Example 3 - Modality classification in biomedical literature: Only fully automatic algorithms are allowed.*	– Fully interactive<br>– Semi-automatic<br>– Fully automatic
10	Organizer participation policy*	Participation policy for members of the organizers' institutes.<br><br>*Example 1 - Brain tumor segmentation: Members of the organizers' institutes may participate but they are not eligible for awards.*<br><br>*Example 2 - Instrument tracking: Members of the organizers' institutes may not participate.*<br><br>*Example 3 - Modality classification in biomedical literature: Members of the organizers' institutes may not participate.*	– Members of the organizers' institutes may participate but they are not eligible for awards and they will not be listed in the leaderboard.<br>– Members of the organizers' institutes may not participate.
11	Training data policy*	Policy on the usage of training data. The data used to train algorithms may, for example, be restricted to the data provided by the challenge or to publicly available data including (open) pre-trained nets.<br><br>*Example 1 - Brain tumor segmentation: The challenge training data may be complemented by other publicly available data.*<br><br>*Example 2 - Instrument tracking: Participants may only use the data provided by the*	– No policy as no training data is required<br>– No additional data allowed<br>– Publicly available data may be added<br>– Private data may be added<br>– Docker container

		challenge for the training of their algorithms.<br><br>*Example 3 - Modality classification in biomedical literature: Participants may use their own data but they have to indicate and describe the additional data.*	
12	Pre-evaluation method	Information on the possibility to evaluate the algorithms before the best runs are to be submitted for an official challenge.<br><br>*Example 1 - Brain tumor segmentation: Results on a pre-test set*<br><br>*Example 2 - Instrument tracking: No pre-evaluation*<br><br>*Example 3 - Modality classification in biomedical literature: No pre-evaluation*	– No pre-evaluation<br>– Private results<br>– Public leaderboard (based on pre-testset)<br>– Results on validation data set
13	Evaluation software	Information on the accessibility of the organizers' evaluation code.<br><br>*Example 1 - Brain tumor segmentation: Software (executable and source code) publicly available from the moment the challenge starts (also after the challenge has ended): <URL>*<br><br>*Example 2 - Instrument tracking: No evaluation software available (Docker concept)*<br><br>*Example 3 - Modality classification in biomedical literature: Software to be used for result submission. Only available for registered participants in the ongoing challenge.*	– Not available<br>– Publicly available: provide URL<br>– Partially available<br>– Available after registration
14	Submission format*	Method that is used for result submission.<br><br>*Example 1 - Brain tumor segmentation: Participants send the algorithm output to the organizers via email.*<br><br>*Example 2 - Instrument tracking: Docker container*<br><br>*Example 3 - Modality classification in biomedical literature: Participants submit a run file that contains their class for each image.*	– Docker container<br>– Cloud<br>– Upload whole code<br>– Upload executable<br>– Send algorithm output to organizers<br>– API<br>– Evaluation Platform
15	Submission instructions	Instructions on how and when the participants should generate and prepare their submissions and what should be included at each stage.<br><br>*Example 1 - Brain tumor segmentation: On <date>, each team has to submit a 2-5 pages short paper with a description of their algorithm and the results on the training and test data sets as described in [ref]. There is no limit in the number of submissions.*<br><br>*Example 2 - Instrument tracking: <link to URL>*<br><br>*Example 3 - Modality classification in biomedical literature: As described in [ref].*	– No instructions<br>– Format of submissions<br>– Timeline<br>– Number of resubmissions allowed<br>– Number of different submissions (different methods) per participant allowed<br>– Missing results/cases allowance<br>– Source code requirement

Mission of the challenge			
16	Field(s) of application*	Medical or biological application that the algorithm was designed for.<br><br>*Example 1 - Brain tumor segmentation: Diagnosis*<br><br>*Example 2 - Instrument tracking: Surgery*<br><br>*Example 3 - Modality classification in biomedical literature: Education*	– Training<br>– Intervention planning<br>– Intervention follow-up<br>– Diagnosis<br>– Screening<br>– Assistance (e.g. tracking tasks)<br>– Research (e.g. cell tracking)<br>– Cross-phase<br>– Education<br>– Prognosis<br>– Prevention<br>– Medical data management
17	Task category(ies)*	Category(ies) of the algorithms assessed.<br><br>*Example 1 - Brain tumor segmentation: Segmentation*<br><br>*Example 2 - Instrument tracking: Localization*<br><br>*Example 3 - Modality classification in biomedical literature: Classification*	– Segmentation<br>– Classification<br>– Tracking<br>– Retrieval<br>– Detection<br>– Localization<br>– Registration<br>– Reconstruction<br>– Modeling<br>– Simulation<br>– Regression<br>– Stitching<br>– Restoration<br>– Prediction<br>– Denoising
18	Target cohort*	Description of subjects/objects from whom the data would be acquired in the final application.<br><br>Remark: A challenge could be designed around the task of medical instrument tracking in robotic kidney surgery. While the validation (see parameter study cohort) could be performed ex vivo in a laparoscopic training environment with porcine organs, the final application (i.e. robotic kidney surgery) would be targeted on real patients with certain characteristics defined by inclusion criteria such as restrictions regarding gender or age.<br><br>*Example 1 - Brain tumor segmentation: Patients diagnosed with glioblastoma that got MRI scans for diagnosis including T1-weighted 3D acquisitions, T1-weighted contrast-enhanced (gadolinium contrast) 3D acquisitions and T2-weighted FLAIR 3D acquisitions.*<br><br>*Example 2 - Instrument tracking: Patients undergoing laparoscopic robotic kidney surgery with the da Vinci Si.*<br><br>*Example 3 - Modality classification in biomedical literature: Biomedical journals from PubMed Central (PMC), i.e. the open access literature indexed in Medline.*	– Healthy volunteers that undergo screening<br>– Patients that undergo laparoscopic surgery<br>– Patients that get an abdominal CT<br>– Patients of a particular database<br>– Patients referred for early Barrett's esophagus cancer without visible abnormalities<br>– Patients attending a state-of-the-art cardiac MRI diagnostic center<br>– Healthy volunteers that are recruited for a certain study<br>– Patients that get chemotherapy<br>– Men with clinical suspicion of having prostate cancer<br>– Standardized cancer cell lines (such as HeLa)<br>– Physicians that use a da Vinci Si for surgical training in an ex vivo setting<br>– OR team (surgeons, nurses, ...) during liver transplantation<br>– Specific journals with an oncology focus (for retrieval tasks)
19	Algorithm target(s)*	Structure/subject/object/component that the algorithm focuses on.<br><br>*Example 1 - Brain tumor segmentation: Glioblastoma*<br><br>*Example 2 - Instrument tracking: Robotic*	– Glioblastoma<br>– Hepatocellular carcinoma (HCC)<br>– Vessels<br>– Liver<br>– Tool tip<br>– (Any) Tumor

		instruments<br>*Example 3 - Modality classification in biomedical literature: Figures showing medical images in the journal*	– Surgeon<br>– Nurse<br>– Specific cell type<br>– Operating room<br>– Specular reflections<br>– Fiber pathway
20	Data origin*	Region(s)/part(s) of subject(s)/object(s) from which the data would be acquired in the final application.<br><br>*Example 1 - Brain tumor segmentation: Brain*<br><br>*Example 2 - Instrument tracking: Abdomen*<br><br>*Example 3 - Modality classification in biomedical literature: JPEG images that appeared in the journal as defined by the target cohort.*	– Abdomen<br>– Liver<br>– Thorax<br>– Whole body<br>– Whole operating room<br>– Cortical gray matter<br>– Specific journal (for retrieval tasks)<br>– Blood obtained from forearm
21	Assessment aim(s)*	Property(ies) of the algorithms aimed to be optimized.<br><br>Remark: Ideally, the metrics used in the study assess the properties of the algorithm as defined by the parameter *assessment aim(s)*. For example, an assessment aim could be targeted on the accuracy of segmentation algorithms. Possible metrics to assess the accuracy include the Dice similarity coefficient (DSC) and the Hausdorff distance (HD).<br><br>*Example 1 - Brain tumor segmentation: Accuracy of enhancing tumor/necrosis/edema segmentation*<br><br>*Example 2 - Instrument tracking: Runtime and robustness*<br><br>*Example 3 - Modality classification in biomedical literature: Accuracy*	– Accuracy<br>– Robustness<br>– Reliability<br>– Precision<br>– Sensitivity<br>– Specificity<br>– Consistency<br>– Runtime<br>– Applicability<br>– Feasibility<br>– Complexity<br>– Usability<br>– User satisfaction<br>– Criteria linked to ergonomics<br>– Integration in (clinical) workflow<br>– Hardware requirements
Study conditions			
22	Study cohort*	Subject(s)/object(s) from whom/which the data was acquired used to validate the algorithm.<br><br>Remark: While a challenge is typically targeted on humans, validation may exclusively involve porcine models or phantoms.<br><br>*Example 1 - Brain tumor segmentation: Patients with glioblastoma (retrospective analysis)*<br><br>*Example 2 - Instrument tracking: Ex vivo porcine organs in a laparoscopic training environment*<br><br>*Example 3 - Modality classification in biomedical literature: PMC journals papers published between 2010 and 2015*	– Specific mouse model<br>– Porcine model<br>– Physical phantom<br>– Patients under controlled conditions<br>– Patients in clinical routine<br>– Porcine liver (in vitro)<br>– In silico data<br>– Healthy volunteers
23	Context information*	Additional information given along with the images. The information may correspond directly to the image data (e.g. tumor volume), to the patient in general (e.g. gender, medical history) or to the acquisition process (e.g. medical device data during endoscopic surgery, calibration data for an image modality).	– No additional information<br>– Genetic information<br>– Age<br>– Gender<br>– Pathology<br>– Clinical diagnoses<br>– Patient number

		Example 1 - Brain tumor segmentation: Clinical patient data: {age, gender, ...}<br><br>*Example 2 - Instrument tracking: API data of robot for each frame and CAD models of instruments*<br><br>*Example 3 - Modality classification for retrieval tasks: None*	– Medical record<br>– Weight<br>– BMI<br>– Race<br>– Cancer (sub-)type<br>– Cancer/disease stage<br>– Body weight/height<br>– Smoking status<br>– Clinical treatment details<br>– Lab data<br>– Clinical history<br>– OR device data<br>– Free text, such as the radiology report, the operation report or histopathology report
24	Center(s)*	Center(s) or institute(s) in which the data was acquired.<br><br>*Example 1 - Brain tumor segmentation: National Center for Tumor Diseases (NCT) Heidelberg*<br><br>*Example 2 - Instrument tracking: As listed on website: <URL>*<br>*Example 3 - Modality classification in biomedical literature: All centers that are mentioned in the articles where the JPEG images originate from*	– Centers involved in the xy study<br>– University Clinic xy<br>– Centers that are part of the xy consortium
24	Imaging modality(ies)*	Imaging technique(s) applied for training/test data acquisition.<br><br>*Example 1 - Brain tumor segmentation: MRI*<br><br>*Example 2 - Instrument tracking: White light endoscopy*<br><br>*Example 3 - Modality classification in biomedical literature: Any medical imaging modality from the following set: <list>*	– Magnetic Resonance Imaging (MRI)<br>– Computed Tomography (CT)<br>– Ultrasound (US)<br>– 3D US<br>– Intravascular US (IVUS)<br>– Positron Emission Tomography (PET)<br>– Light Microscopy (LM)<br>– Electron Microscopy (EM)<br>– X-Ray<br>– Optical Coherence Tomography (OCT)<br>– Endomicroscopy (w/ or w/o dye)<br>– SPECT<br>– Video<br>– Fluoroscopy<br>– Thermography
26	Acquisition device(s)	Device(s) used to acquire the validation data. This includes details on the device(s) used to acquire the imaging data (parameter imaging modality(ies)) as well as information on additional devices used for validation (e.g. tracking system used in a surgical setting):<br><br>*Example 1 - Brain tumor segmentation: 3 T Philips Achieva scanner and GE Signa 1.5 T scanner*<br><br>*Example 2 - Instrument tracking: da Vinci Si endoscope and NDI Aurora electromagnetic tracking system with standard electromagnetic field generator*<br><br>*Example 3 - Modality classification in biomedical literature: Large variety of devices provided as a list*	– Philips Gyro Scan NT 1.5 Tesla scanner<br>– GE Discovery ST multislice PET/CT scanner<br>– G-EYE Videocolonoscope<br>– MINDRAY DC-30 US scanner<br>– NDI Aurora electromagnetic tracking system with Tabletop field generator<br>– None (e.g. in case of in silico validation)<br>– Unknown (e.g. in case of retrieval tasks)
27	Acquisition protocol(s)	Relevant information on the imaging process/ data acquisition for each acquisition device.	– Dimension (e.g. 2D, 3D+t)<br>– Timepoints<br>– Position and orientation of patient

		Example 1 - Brain tumor segmentation: T1-weighted 3D acquisitions (1.0 x 1.0 x 1.0 mm^3), T1-weighted contrast-enhanced (gadolinium contrast) 3D acquisitions (1.0 x 1.0 x 1.0 mm^3), T2-weighted FLAIR 3D acquisitions (0.9 x 0.9 x 2.0 mm^3)<br><br>*Example 2 - Instrument tracking: Only left frame recorded with da Vinci Si system*<br><br>*Example 3 - Modality classification in biomedical literature: Generally unknown*	– Radiation dose<br>– Radiopharmaceuticals<br>– Frequency of imaging<br>– Resolution<br>– Pixel spacing<br>– MRI: T1, T2, contrast enhanced, ...<br>– MRI: Repetition time (TR), echo time (TE)<br>– Field of view (FOV)<br>– Post-processing<br>– Employed dye(s)<br>– Use of filtering<br>– LM: phase contrast (PhC)<br>– CT: kEV<br>– Microscopy: Stainings<br>– Contrast agents
28	Operator(s)	Characteristics of operator(s) involved in the data acquisition process.<br><br>*Example 1 - Brain tumor segmentation: N/A*<br><br>*Example 2 - Instrument tracking: 2 male surgeons with more than 10 years of experience in laparoscopic surgery*<br><br>*Example 3 - Modality classification in biomedical literature: Unknown*	– Surgeon<br>– Engineer<br>– Nurses<br>– Robot<br>– Patient (e.g. with a smartphone app used for melanoma detection)<br>– OR<br>– Technician<br>– Medical trainee<br>– Biologist<br>– Radiologist<br>– Medical Physicist<br>– Radiographer<br>– Sonographer<br>– Unknown<br><br>Reported information may be:<br>– Number<br>– Function<br>– Names<br>– Skill level (e.g. measured in the number of years of experience)
Challenge data sets			
29	Distribution of training and test cases*	Describes how training and test data were split and for what reason this division was chosen. This should include information (1) on why a specific proportion of training/test data was chosen, (2) why a certain total amount of cases was chosen and (3) why certain characteristics were chosen for the training/test set (e.g. class distribution according real-world distribution vs equal class distribution).<br><br>*Example 1 - Brain tumor segmentation: 80% training data and 20% test data according to common practice in machine learning.*<br><br>*Example 2 - Instrument tracking: All video sequences that are publicly available from site xy. Video sequences from institution x (50%) used for training. Video sequences from institution y (50%) used for testing.*<br><br>*Example 3 - Modality classification in biomedical literature: 60% training data and 40% test data according to common practice in the domain [ref]. Random assignment of images to training/test data sets.*	– Not applicable as no training data is provided<br>– Randomly distributed<br>– Balanced false and negative cases<br>– 80% training data, 20% test data as recommended by [ref]

30	Category of training data generation method*	Method for determining the desired algorithm output for the training data. Possible methods include manual image annotation, in silico ground truth generation and annotation by automatic methods, and no training data generated.<br><br>*Example 1 - Brain tumor segmentation: Hybrid: Initiation by algorithm and refinement/correction by expert physician*<br><br>*Example 2 - Instrument tracking: Crowdsourced annotations*<br><br>*Example 3 - Modality classification in biomedical literature: Manual annotation*	– Ground truth from simulation (exact)<br>– Reference from algorithm<br>– Reference from single human rater<br>– Reference from multiple human raters<br>– Hybrid: Initiation by algorithm, refinement by expert physician<br>– Reference derived from clinical practice (diagnosis/disease code etc.)<br>– Crowdsourced annotations
31	Number of training cases*	Number of cases that can be used for algorithm training and parameter optimization. A case encompasses all data that is processed to produce one result (e.g. one segmentation) as well as the corresponding reference result.<br><br>*Example 1 - Brain tumor segmentation: 400*<br><br>*Example 2 - Instrument tracking: 5 video sequences, each containing 100 annotated frames*<br><br>*Example 3 - Modality classification in biomedical literature: 6,000*	– No training data provided<br>– 100 images<br>– 100 raw endoscopic video sequences with a total of 1,000 fully annotated frames
32	Characteristics of training cases*	Additional information on the training cases describing their nature, such as the level of detail of the annotations (e.g. fully vs weakly annotated).<br><br>*Example 1 - Brain tumor segmentation: Pixel-level segmentation of the structures of interest and additional clinical information as described in context information.*<br><br>*Example 2 - Instrument tracking: Full segmentation of 100 frames (equally distributed) in each video sequence. No segmentation of the instruments in the remaining frames, but API information on instrument poses available for all frames.*<br><br>*Example 3 - Modality classification in biomedical literature: Full annotation - modality/image type per image*	– No training data provided<br>– Full annotation (pixel level)<br>– Weak annotation (image level): tumor volume, disease stage<br>– Mixed annotation: 1,000 fully annotated images, 100 weakly annotated images<br>– 100 endoscopic video images with 10 fully annotated training images
33	Annotation policy for training cases*	Instructions given to the annotators prior to training case annotation. This may include description of a training phase with the software.<br><br>*Example 1 - Brain tumor segmentation: The annotator was instructed to segment the edema using the T2 and FLAIR images. The enhancing tumor was subsequently to be segmented on the T1 contrast-enhanced modality. Finally, the necrotic core was to be outlined using the T1 and contrast-enhanced T1 image. The annotations were to be performed in axial slices. The undergraduate student received training on 5 cases (by the radiologist) to extract the weak labels (see parameter: context information).*	– Challenge-specific detailed instructions – e.g. should an annotation be performed along a tumor boundary or including a safety zone? Is it allowed to guess a boundary if not clearly visible?<br>– URL to annotation instructions<br>– What tissue would you resect?<br>– Where would you take a (small) biopsy?

		Example 2 - Instrument tracking: <URL to annotation instructions><br><br>*Example 3 - Modality classification in biomedical literature: Instruction to label each cropped image with the modality that has (presumably) been used to acquire the image shown in the figure. List of potential labels: <list>. No further instructions, no training.*	
34	Annotator(s) of training cases*	Details on the subject(s)/algorithm(s) who/which annotated the training data.<br><br>*Example 1 - Brain tumor segmentation: Weak annotation (parameter context information) extracted from medical reports by undergraduate medical student; full image annotation performed by radiologist.*<br><br>*Example 2 - Instrument tracking: Crowdsourcing of image annotations on the platform Amazon Mechanical Turk according to the method in paper [ref]. Pose data (parameter context information) is automatically acquired by the robot*<br><br>*Example 3 - Modality classification in biomedical literature: Three PhD students*	– No training data provided<br>– Surgeon who has done >100 cases of a specific type of surgery<br>– Undergraduate physician (third year)<br>– Engineer who developed the software<br>– Physician with no prior experience in usage of the software<br>– Crowd<br>– Algorithm xy
35	Annotation aggregation method(s) for training cases*	Method(s) used to merge multiple annotations for one case.<br><br>*Example 1 - Brain tumor segmentation: (only one observer)*<br><br>*Example 2 - Instrument tracking: According to [ref]*<br><br>*Example 3 - Modality classification in biomedical literature: Majority vote*	– No aggregation<br>– Simultaneous Truth and Performance Level Estimation (STAPLE)<br>– Majority vote<br>– An additional annotator resolves conflicts<br>– Average<br>– Selective and Iterative Method for Performance Level Estimation (SIMPLE)<br>– Level-set based approach maximizing the a posteriori probability (LSML)<br>– Strict combination (positive if and only if all annotators agree)<br>– No training data is required
36	Category of test data generation method*	Method for determining the reference (i.e. the desired algorithm result, also referred to as gold standard) which is used for assessing the participants' algorithms. Possible methods include manual image annotation, in silico ground truth generation and annotation by automatic methods.<br><br>*Example 1 - Brain tumor segmentation: Manual annotation*<br><br>*Example 2 - Instrument tracking: Crowdsourced annotations*<br><br>*Example 3 - Modality classification in biomedical literature: Manual annotation by multiple observers*	– Ground truth from simulation (exact)<br>– Reference from algorithm<br>– Reference from single human rater<br>– Reference from multiple human raters<br>– Acquired through previously validated methods according to [ref]<br>– Reference derived from clinical practice (diagnosis/disease code etc.)<br>– Crowdsourced annotations<br>– Hybrid methods (e.g. initiation by algorithm, refinement by expert physician)
37	Number of test cases*	Number of cases used to assess the performance of an algorithm. A case encompasses all data that is processed to produce one result as well as the corresponding reference result (typically not provided to the participants).	– 100 images<br>– 100 raw endoscopic video sequences with a total of 1,000 fully annotated frames

		Example 1 - Brain tumor segmentation: 100<br><br>*Example 2 - Instrument tracking: 5 video sequences, each containing 100 annotated frames.*<br><br>*Example 3 - Modality classification in biomedical literature: 4,000*	
38	Characteristics of test cases*	Additional information on the test cases describing their nature, such as the level of detail of the annotations (e.g. fully vs weakly annotated).<br><br>*Example 1 - Brain tumor segmentation: Pixel-level segmentation of the structures of interest and additional clinical information as described in context information*<br><br>*Example 2 - Instrument tracking: Full segmentation of 100 frames (equally distributed) in each video sequence. No segmentation of the instruments in the remaining frames, but API information on instrument poses available for all frames*<br><br>*Example 3 - Modality classification in biomedical literature: Full annotation - modality/image type per image*	– Full annotation (pixel level)<br>– Weak annotation (image level): tumor volume, disease stage<br>– Mixed annotation: 1,000 fully annotated images, 100 weakly annotated images<br>– 100 endoscopic video images with 10 fully annotated test images
39	Annotation policy for test cases*	Instructions given to the annotators prior to test case annotation. This may include description of a training phase with the software.<br><br>*Example 1 - Brain tumor segmentation: The annotator was instructed to segment the edema using the T2 and FLAIR images. The enhancing tumor was subsequently to be segmented on the T1 contrast-enhanced modality. Finally, the necrotic core was to be outlined using the T1 and contrast-enhanced T1 image. The annotations were to be performed in axial slices. The undergraduate student had received training on extracting the weak labels when annotating the training images.*<br><br>*Example 2 - Instrument tracking: <URL to annotation instructions>*<br><br>*Example 3 - Modality classification in biomedical literature: Instruction to label each cropped image with the modality that has (presumably) been used to acquire the image shown in the figure. List of potential labels: {...}. After two observers have annotated the images independently, the cases where the annotators disagreed will be automatically retrieved. These should be shown to the third observer for resolving conflicts. Training on the training cases.*	– Challenge-specific detailed instructions – e.g. should an annotation be performed along a tumor boundary or including a safety zone? Is it allowed to guess a boundary if not clearly visible?<br>– URL to annotation instructions<br>– What tissue would you resect?<br>– Where would you take a (small) biopsy?
40	Annotator(s) of test cases*	Details on the subject(s)/algorithm(s) who/which annotated the test data.<br><br>*Example 1 - Brain tumor segmentation: Radiologist with 5 years of experience*<br><br>*Example 2 - Instrument tracking: Crowdsourcing*	– Surgeon who has done >100 cases of a specific type of surgery<br>– Undergraduate physician (third year)<br>– Engineer who developed the software<br>– Physician with no prior experience in usage of the software

		on the platform Amazon Mechanical Turk according to the method [ref]<br><br>*Example 3 - Modality classification in biomedical literature: Two PhD students and a radiologist*	– Crowd<br>– Algorithm xy
41	Annotation aggregation method(s) for test cases*	Method(s) used to merge multiple annotations for one case (if any).<br><br>*Example 1 - Brain tumor segmentation: No merging*<br><br>*Example 2 - Instrument tracking: According to [ref]*<br><br>*Example 3 - Modality classification in biomedical literature: Expert resolves conflicts*	– No aggregation (ranking provided for each annotator)<br>– STAPLE<br>– Majority vote<br>– An additional annotator resolves conflicts<br>– Average<br>– SIMPLE<br>– LSML<br>– Strict combination (positive if and only if all annotators agree)
42	Data pre-processing method(s)	Methods used for pre-processing the raw data before it is provided to the participants.<br><br>*Example 1 - Brain tumor segmentation: Registration of different contrasts using algorithm x and denoising using algorithm y. Both performed in framework z. Resampling to common coordinate system with spatial resolution 1x1x1mm^3; skull stripping according to [ref], bias field correction according to [ref].*<br><br>*Example 2 - Instrument tracking: Irrelevant scene removal using algorithm xy [ref] with parameters z.*<br><br>*Example 3 - Modality classification in biomedical literature: Cropping to figures; if compound figures are included, then these are separated manually into the subfigures using the software xy.*	– No pre-processing steps<br>– Registration with a particular method<br>– Segmentation with a particular method<br>– Resampling of raw data<br>– Re-orientation<br>– Normalization<br>– Data cleaning<br>– Instance selection<br>– Feature extraction<br>– Feature selection<br>– Video anonymization<br>– Bias correction<br>– Intensity standardization<br>– White balancing<br>– Smoothing<br>– Skull stripping<br>– Histogram Matching<br>– Background subtraction<br>– Uneven background intensity correction<br>– Image enhancement (contrast/brightness change, histogram equalization)<br>– Data format conversion (DICOM to NIFTI)<br>– Journals: cropping, grey level reduction
43	Potential sources of reference errors	Most relevant possible error sources related to the estimation of the reference. This may include errors related to the image acquisition method, user errors and errors resulting from the pre-processing method applied. It may be quantified by inter- and intra-observer variability, for example. This information must be provided for the test cases and may be provided for the training cases.<br><br>*Example 1 - Brain tumor segmentation: Tissue classes (tumor necrosis, enhancing tumor and edema) difficult to distinguish. Previous studies suggest inter-rater disagreement of xy [ref].*<br><br>*Example 2 - Instrument tracking: The provided API robot pose data has an estimated accuracy of x according to additional experiments performed with the calibration phantom described in [ref]. The accuracy of crowdsourced segmentations of medical instruments with the applied method has been estimated to be around z [ref].*<br><br>*Example 3 - Modality classification in biomedical*	Sources of error:<br>– Partial volume effects<br>– Errors resulting from surface generation<br>– User errors<br>– Organ deformation<br>– Distortion of electromagnetic field<br>– Noise<br>– Imaging system aberrations (deteriorated point spread function resulting in blurring)<br>– Imaging system artifacts (dust, motion, spikes, etc.)<br>– Interlacing<br><br>Quantification:<br>– Inter-/ intra-observer variability<br>– Confidence intervals<br>– Kappa statistics<br>– Correlation coefficients<br>– Signal-to-noise ratio<br>– Resolution<br>– Bland-Altman Plots

		literature: Previous experiments [ref] suggest that the main source of error is related to ambiguity in the images, as there is an extremely large variability and sometimes the defined image types are hard to fit into the existing hierarchy of a small number of image types; other mistakes can be linked to annotating quickly and not looking at sometimes fine differences. Inter-observer variability in a separate experiment was x%.	
Assessment method			
44	Metric(s)*	Function(s) to assess a property of an algorithm. These functions should reflect the validation objective (see parameter *assessment aim(s)*).<br><br>*Example 1 - Brain tumor segmentation: 95% HD and precision applied separately to necrosis, enhancing tumor and edema.*<br><br>*Example 2 - Instrument tracking: Runtime per frame on a xy device; Percentage of frames with DSC below threshold t.*<br><br>*Example 3 - Modality classification in biomedical literature: Accuracy*	– Hausdorff distance (HD)<br>– Dice similarity coefficient (DSC)<br>– Jaccard index<br>– Computation time<br>– Recall<br>– Precision<br>– Area under curve (AUC)<br>– Root mean square error (RMSE)<br>– Absolute volume difference<br>– True positive rate<br>– Computational complexity<br>– Average symmetric surface distance (ASSD)<br>– F1-Score<br>– Specificity<br>– Intraclass correlation coefficient<br>– Concordance index<br>– MAP
45	Justification of metric(s)*	Justification why the metric(s) was/were chosen, preferably with reference to the clinical application.<br><br>*Example 1 - Brain tumor segmentation: 95% Hausdorff Distance as opposed to standard HD: Try to avoid that outliers have too much weight. All other metrics are commonly used in segmentation assessment (cf. ref. xy).*<br><br>*Example 2 - Instrument tracking: Thresholded DSC according to best practice recommendations [ref]. Computation time as clinical application requires video rate performance.*<br><br>*Example 3 - Modality classification in biomedical literature: Accuracy is the most common method as easy to interpret; geometric mean assures that all classes are well classified and not only the majority classes.*	– According to best practice recommendations [ref]<br>– According to paper [ref]
46	Rank computation method*	Method used to compute a rank for all participants based on the generated results on the test data. It may include methods for aggregating over all test cases and/or for determining a final rank from multiple single metric-based ranks. It also includes the ranking order for tied positions.<br><br>*Example 1 - Brain tumor segmentation: For each participant pi and each test case cj: Compute the metric values for the 95% Hausdorff distance and precision. For each participant pi and each test case cj, determine the rank corresponding to both metrics (i.e.*	Example 1:<br>0. Initialization: For each participant pi and each test case cj: compute metric values M1(pi,cj) and M2(pi,cj) for metrics M1 and M2.<br>1. Metric-based aggregation: For each participant pi compute the median over all cases cj for each metric M1(pi) and M2(pi).<br>2. For each participant pi, compute the sum over the two metrics as M1(pi) + M2(pi).<br>3. Build rank for each participant by sorting the values M1(pi) + M2(pi) for each participant.

		R(precision, pi, cj): descending order for precision, R(HD, pi, cj) ascending for 95% HD). For each participant pi and each test case cj, compute the average rank R(pi, cj) over both metric ranks. Finally, compute the average over all case-specific ranks to get one final rank for each participant pi.<br><br>*Example 2 - Instrument tracking: For each participant pi compute the average metric value for the thresholded DSC. Build the rank for each participant by sorting the accumulated metric values. In case of tied positions, perform the ranking according to computation times.*<br><br>*Example 3 - Modality classification for retrieval tasks: Ranking performed according to [ref].*	Example 2:<br>0. Initialization: For each participant pi and each test case cj: compute metric values M1(pi,cj), M2(pi,cj) and M3(pi,cj) for metrics M1-M3.<br>1. Case-based aggregration: For each partipant pi and each case cj, determine the performance score sj on case cj: sij := 1/3 (M1(pi,cj) + M2(pi,cj) + M3(pi,cj)).<br>2. For each partipant pi and each case cj, determine the rank R(pi,cj) for case cj according to score sj.<br>3. Compute the average over all case-specific ranks for each participant pi si := 1/N*Sum_j (R(pi,cj)) to obtain the final rank.
47	Interaction level handling*	Method(s) used to handle any diversity in the level of user interaction when generating the performance ranking.<br><br>*Example 1 - Brain tumor segmentation: Weighting function (automatic methods are ranked higher)*<br><br>*Example 2 - Instrument tracking: Only automatic algorithms are allowed.*<br><br>*Example 3 - Modality classification in biomedical literature: Only automatic methods are allowed.*	– Indication in ranking<br>– Separate ranking for fully-automatic methods<br>– Only automatic methods allowed
48	Missing data handling*	Methods used to manage submissions with missing results on test cases.<br><br>*Example 1 - Brain tumor segmentation: In case of missing data for participant pi and case cj, the case-based ranks for all metrics m R(m, pi, cj) are set to the maximum.*<br><br>*Example 2 - Instrument tracking: Only complete submissions are evaluated.*<br><br>*Example 3 - Modality classification in biomedical literature: Missing results are considered to be incorrectly classified*	– Missing data not allowed (incomplete submissions not evaluated)<br>– Missing data ignored<br>– Missing data handled as in [ref]
49	Uncertainty handling*	Method(s) used to make uncertainties in ranking explicit.<br><br>*Example 1 - Brain tumor segmentation: Test the sensitivity of the ranking with bootstrapping according to [ref].*<br><br>*Example 2 - Instrument tracking: Test the sensitivity of a ranking by leaving out different amounts of test data as described in [ref].*<br><br>*Example 3 - Modality classification in biomedical literature: None*	Test sensitivity of the ranking by<br>– Leaving out test data<br>– Bootstrapping approaches<br>– Changes in rank computation details<br>– Changes in reference annotation
50	Statistical test(s)*	Statistical test(s) used to compare the results of challenge participants.<br><br>*Example 1 - Brain tumor segmentation: T-test used to test the stability of the first three ranks as described in [ref].*	Quantities on which the hypothesis is taken:<br>– Stability of the ranking<br>– See whether the best results have statistically significant differences<br><br>Tests:<br>– Wilcoxon-Mann-Whitney test

		Example 2 - Instrument tracking: U-test used to test statistically significant differences between the participants as described in [ref].<br>*Example 3 - Modality classification in biomedical literature: No statistical tests.*	– t-test (paired, unpaired, one-sided, two-sided)<br>– Saphiro-Wilk test
Challenge outcome			
51	Information on participants	Information on participating teams including affiliation and specifics of competing algorithm, preferably with reference to a document.	Should include:<br>– Acronym<br>– Affiliation<br>– Contact person<br>– Team members<br>– Method description including parameter instantiation<br>– Submission/attempt number<br>– Relevant reference publication
52	Results	Values of all metrics and rankings including the number of submissions for each participant.<br><br>*Example 1 - Brain tumor segmentation: Not yet available*<br><br>*Example 2 - Instrument tracking: Matrix with colums = participants; rows = (case j, metric k); in addition two rankings for the two metrics and the final ranking.*<br><br>*Example 3 - Modality classification in biomedical literature: Two rankings corresponding to the two metrics and the associated aggregated values.*	– Not yet available<br>– Link to URL<br>– Matrix with results for each participant, each metric and each test case plus ranking
53	Report document	Evaluating and summarizing information about the challenge or the workshop published in a scientific journal or similar literature, preferably with DOI.<br><br>*Example 1 - Brain tumor segmentation: IEEE Transactions on Medical Imaging: <link to pdf>*<br><br>*Example 2 - Instrument tracking: arXiv publication: <URL>*<br><br>*Example 3 - Modality classification in biomedical literature: Publication in CEUR: <DOI>*	– No publication<br>– DOI<br>– Link to document<br>– Full citation<br>– arXiv ID

Parameter list for biomedical challenge design.
**: Parameters used for structured challenge submission for the MICCAI 2018 challenges.*

Supplementary Table 3: Best practice recommendations

Problem	Best practice recommendation(s)	Open research question(s)
Incomplete reporting	Instantiate the full parameter list (Table 1) when reporting on a challenge to maximize transparency, interpretability and reproducibility.<br><br>Publish a peer-reviewed report on the challenge.	How to describe the data in a structured and standardized manner (e.g. using ontologies)? [84-87]
Unclear challenge goal	Define a relevant, specific and feasible goal which the challenge will address.<br><br>Decide on whether to perform an *insight challenge*, the objective of which is to gain insight into a problem and potentially identify a research direction, or a *deployment challenge,* the objective of which is to solve a problem and identify the best-performing algorithms based on a huge benchmarking set.<br><br>Decide whether the challenge should be competitive (with one winner?) or collaborative or combining elements of both.	How to judge the utility (scientific advancement, clinical relevance, biological or clinical insights, implications for patient care, commercial readiness) of a challenge? [88-91]<br><br>For *deployment challenges*, how to determine feasibility of clinical deployment in the near, medium and long term? [92-94]<br><br>How to provide incentives for participating in collaborative challenges [95-96, 145]?
Lack of representative-ness	Use data from multiple sources (e.g. sites, devices).<br><br>Ensure that the selected data collection covers the natural variability of imaged objects.<br><br>Be aware of the effects of imbalanced training data [97] when designing the training data set.<br><br>Be aware that many methods require the training and test data to have comparable distributions.	How to determine the required number of training/test cases for a given task? [98-103]<br><br>How to avoid bias in the training/test data? [86, 104-107]<br><br>How to design a challenge that covers the heterogeneity of clinical practice?
Low annotation quality	Use multiple annotators per test case.<br><br>Provide clear guidelines for the annotators.<br><br>Choose the tools for speeding up annotations carefully as they may lead to bias in the annotations (cf. e.g. [108]).<br><br>Find a good compromise between quantity and quality. Consider maximizing annotation quality for the test data ('gold corpus') while increasing quantity at the expense of quality in the training data ('silver corpus'). In this case, be aware of the different distributions of training and test data.<br><br>Assign certified physicians with standardized training a key role in imaging data annotation to maximize inter-reader agreement [26-28]	How to choose the number of observers for a specific task? [112-115]<br><br>How to best combine multiple annotations? [116-120]<br><br>How to represent, quantify and compensate uncertainty in annotations? [121-122]<br><br>How to provide incentives (especially for clinicians) for data acquisition and annotation? [123]<br><br>How to make data annotation more efficient? [104-105, 108, 124]
Suboptimal metric(s)	Make sure the metrics reflect the challenge goal.<br><br>Choose metrics that capture the clinically/biologically relevant differences.<br><br>Be aware of metric-specific biases in favor of/against various properties [125].<br><br>In segmentation challenges, be aware (1) that the DSC yields more robust rankings than the HD and (2) that the HD yields more robust rankings than the HD95 (Fig. 3).<br><br>Consider including supplementary *usability* metrics related to computation time, memory consumption, number of supported platforms and number of parameters, etc. [126].	How to determine the best (variant of a) metric or a set of metrics for a given task? [127-129]<br><br>How to better consider clinical relevance in the performance metrics (e.g. by having radiologists quantify the negative effect of segmentation errors)?

Poor ranking schemes	Be aware of mutually dependent metrics [129].<br><br>Ensure robust rankings.<br>- Perform metric-based aggregation rather than case-based aggregation to obtain more robust rankings (Fig. 2).<br>- Use the mean rather than the median to obtain more robust rankings in aggregation-based ranking (Fig. 2).<br>- Consider alternative ranking schemes that have a higher tendency to group algorithms compared to aggregation-based methods using the mean or median of metric values [47-48] (see Supplementary Discussion).<br><br>Develop a strategy to handle missing values (cf. Supplementary Discussion2).<br><br>When applying case-based rankings, consider the tradeoff between robustness (Fig. 2) and good missing value handling.<br><br>Be aware that a statistically significant difference in a metric value may not be clinically/biologically relevant. Vice versa, a clinically relevant difference in performance may not be statistically significant due to small sample size.<br><br>Report multiple metric results and provide appropriate visualizations to highlight strengths and weaknesses of different methods [132-134].	How to handle missing data when aggregating metric values?<br><br>How to determine an appropriate ranking scheme for a given application? [135-138]<br><br>How to group algorithms (i.e. assign the identical rank) in a sensible manner? [137-139]
Poor uncertainty handling	Quantify the uncertainties of annotations and rankings and make them explicit:<br>- Report inter-observer variability for reference annotations.<br>- Perform bootstrapping to quantify ranking stability (cf. Fig. 3).<br><br>Consider generating fuzzy (probabilistic) reference data and allowing submission of fuzzy results [118, 140].	How to incorporate known uncertainties in the reference annotations in the metric computation? [122]<br><br>How to quantify the uncertainty of a ranking? [136-138]
Cheating and overfitting	Publish the challenge design before the challenge according to the parameters in Table 1.<br><br>Aim for Docker-based solutions [141] or on-site challenges to reduce the risk of cheating.<br><br>Otherwise release more test cases than are used for validation (keep the real ones for which annotations are available confidential).<br><br>Do not participate in your own challenge, or otherwise, make the participation transparent.<br><br>Encourage open source release of the algorithms' code.<br><br>Ensure a possibility to deploy/execute the winning algorithm.	What is a good lifecycle for a challenge (considering both the dynamics of algorithm development and the overfitting problem)? [104-105, 142-144]
Infrastructural hurdles	Use a web-based platform to run the challenge (e.g. [146-149]).<br><br>Consider cloud-based infrastructure for huge data sets and computationally demanding tasks (e.g. [150]).<br><br>Choose or define a suitable algorithm output format and provide tools for the computation of metrics in this format. Include sample algorithm(s)/workflow(s). In general, 16-bit formats are preferred (e.g. DICOM/TIFF or HDF5)	How to establish globally respected standards?

Problems related to current challenge design and organization, best practice recommendations for them and open research challenges (including literature for further reading) relating to them.

Supplementary Discussion

This material presents and discusses three methods for computing ranks: (1) Metric-based aggregation, the most widely used method, (2) case-based aggregation, the 2nd most commonly used ranking scheme, and (3) significance ranking, a scheme that the authors consider a promising alternative when requiring straightforward missing value handling and the natural assignment of identical ranks to algorithms that show only marginal differences. Note that we present the most commonly applied variants of the ranking schemes. A multitude of variations of these three ranking methods are conceivable.

Metric-based aggregation

The principle of metric-based aggregation is illustrated in Supplementary Discussion Fig. 1.

For each algorithm a_i, $i = 1, ..., n$:

1. Determine the performance $m_k(a_i, t_j)$ for each test case t_j, $j = 1, ..., m$ and each metric m_k, $k = 1,..., o$
2. If $m_k(a_i, t_j)$ = N/A, set $m_k(a_i, t_j)$ to the worst possible value of m_k (e.g. 0 in the case of the DSC) or a "punishing value" if the metric is not bounded.
3. Aggregate metric values $m_k(a_i, t_j)$, $k=1, ..., o$ over all test cases t_j, $j = 1, ..., m$ (e.g. with the mean or median) to obtain a score for each metric $m_k(a_i)$.
4. Aggregate over all metrics (possibly in a weighted manner) to obtain a score for each algorithm $s(a_i)$.

Compute the rank for each algorithm $r(a_i)$ based on the scores $s(a_i)$, $i = 1, ..., n$.

Note that aggregation may alternatively be performed over the metric values first, as illustrated in Supplementary Discussion Fig. 1b.

Metric-based aggregation is extremely intuitive to interpret and – according to our analysis – more robust than the 2nd most commonly used ranking scheme (case-based aggregation). Another major strength is that the incorporation of a new algorithm to an existing ranking is straightforward as it only requires the metric values for the method to be added. This becomes important when using challenge data sets as benchmarks after the challenge has been run because a comparison to existing methods is possible without having access to the source code or individual test case results.

One of the most severe problems with metric-based aggregation is that missing data handling is not straightforward when metrics are not bounded. When using a "punishing value" to represent N/As, the mean determined over a set of cases depends on this value and so does the ranking (potentially). Furthermore, arbitrarily small differences in aggregated metric values result in different ranks. This issue cannot easily be solved with statistical significance tests because pairwise comparisons may result in inconsistent rankings.

(a) Aggregate over all test cases first

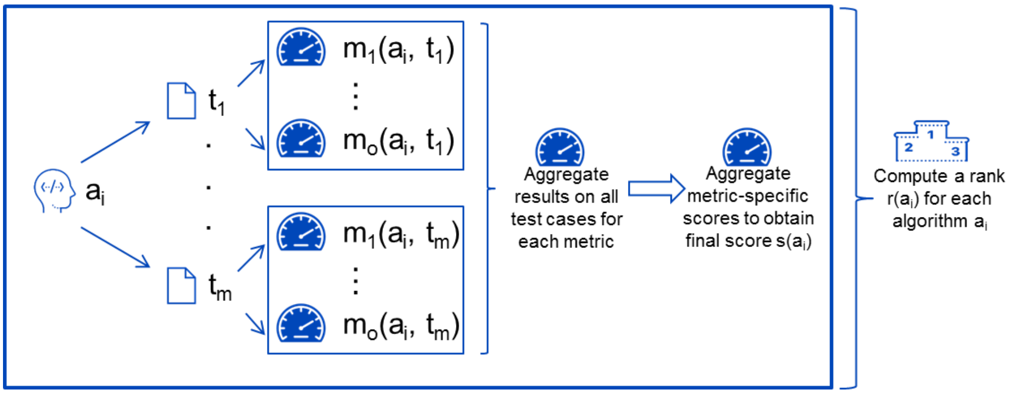

(b) Aggregate over metric first

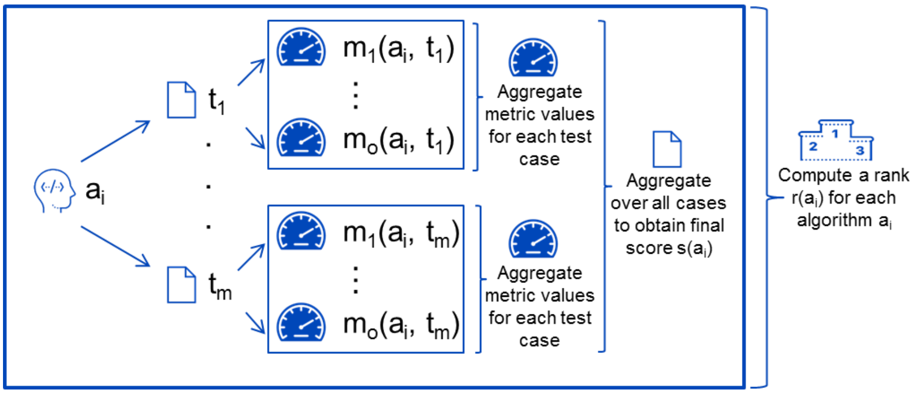

Supplementary Discussion Fig. 1: Two variants of metric-based aggregation. A_i: Algorithm i, i = 1, …, n, t_j: test case j, j = 1, …, m, m_k: metric k, k = 1, …, o. Icons by https://icons8.com/.

Case-based aggregation

The principle of case-based aggregation is illustrated in Supplementary Discussion Fig. 2.

For each test case t_j, $j = 1, ..., m$:

1. Determine the performance $m_k(a_i, t_j)$ for each algorithm a_i, $i = 1, ..., n$ and each metric m_k, $k = 1, ..., o$
2. Based on $m_k(a_i, t_j)$, compute a metric-specific and test case-specific rank $r_{k,j}(a_i)$ for each algorithm a_i. If $m_k(a_i, t_j)$ = N/A, $r_{k,j}(a_i)$ is set to the worst possible rank.
3. Compute an overall rank $r_j(a_i)$ for each test case by aggregating (possibly in a weighted manner) over metric ranks $r_{k,j}(a_i)$ (e.g. using the mean or the median).

Compute the final rank $r(a_i)$ by aggregating over test cases $r_k(a_i)$, $i = 1, ..., n$ (e.g. using the mean or the median).

The key advantage of case-based aggregation is that missing data handling is straightforward. Also, because the aggregation is performed over discrete values, the grouping of similarly performing algorithms is more likely compared to metric-based aggregation. On the other hand, our analysis suggests that case-based aggregation is less robust than metric-based aggregation. Furthermore, the dynamic adding of a new algorithm to the ranking requires access to all performance data (metric outputs for all algorithms on all cases).

(a) Aggregate over all test cases first

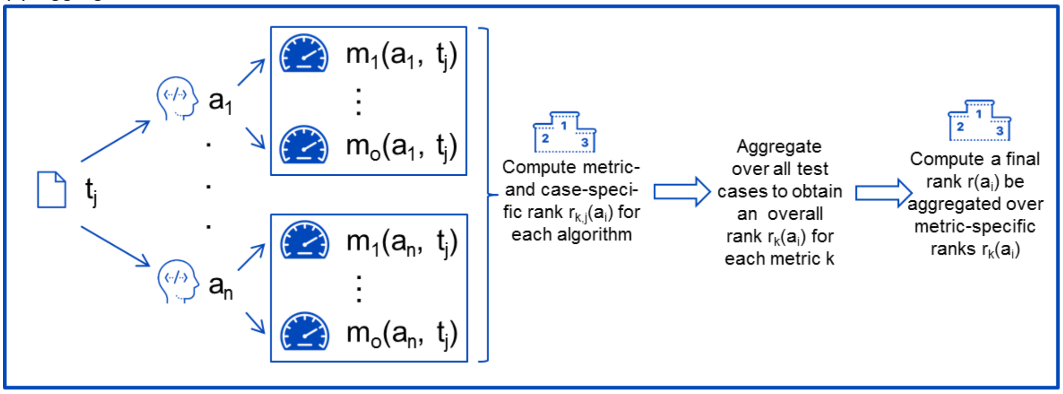

(b) Aggregate over metric first

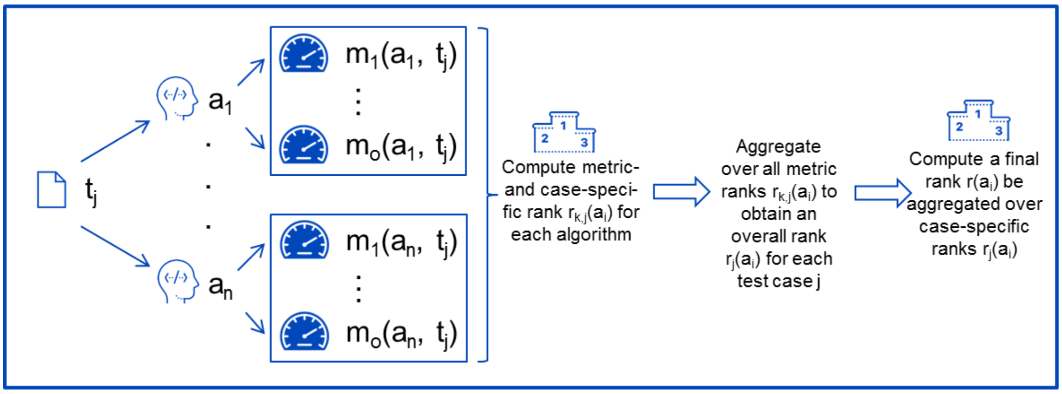

Supplementary Discussion Fig. 2: Case-based aggregation. A_i: Algorithm i, i = 1, …, n, t_j: test case j, j = 1, …, m, m_k: metric k, k = 1, …, o. Icons by https://icons8.com/.

Significance ranking

The principle of significance ranking is illustrated in Supplementary Discussion Fig. 3.

Select a significance level α, e.g. α = 5%.
For each metric m_k, $k = 1, ..., o$:

1. Determine performance $m_k(a_i, t_j)$ of each algorithm a_i, $i = 1, ..., n$ for each test case t_j, $j = 1, ..., m$
2. Perform all pairwise comparisons between algorithms $(a_i, a_{i'})$ with the values $m_k(a_i, t_j)$ and $m_k(a_{i'}, t_j)$ using Wilcoxon signed rank test (with α)
3. Determine a significance score $s_k(a_i)$, $i = 1, ..., n$ which equals the number of algorithms performing significantly worse than a_i according to the test
4. Compute the ranking (shared ranks possible) based on the scores $s_k(a_i)$, $i = 1, ..., n$ with the highest score corresponds to the best algorithm(s) (rank 1)

The final ranking over all metrics is computed by aggregating the significance scores over all metrics by mean or median, or, if o is sufficiently large, by application of the significance ranking algorithm to the significance scores for all metrics.

The selection of the significance level α should depend on the number of test cases (m). While α can be selected as a small value (e.g. 1% or 0.1%) when m is large, a small α for small m will lead to large groups of algorithms sharing the same rank because no significant differences are found in pairwise comparisons of algorithms.

The key advantage of this method is that it naturally results in shared places when performance differences are minor. Also, missing value handling is better compared to metric-based aggregation. On the other hand, the ranking scheme is more complex to implement compared to popular alternatives. Also, the dynamic adding of a new algorithm to the ranking is not straightforward as the significance scores of all algorithms may potentially change.

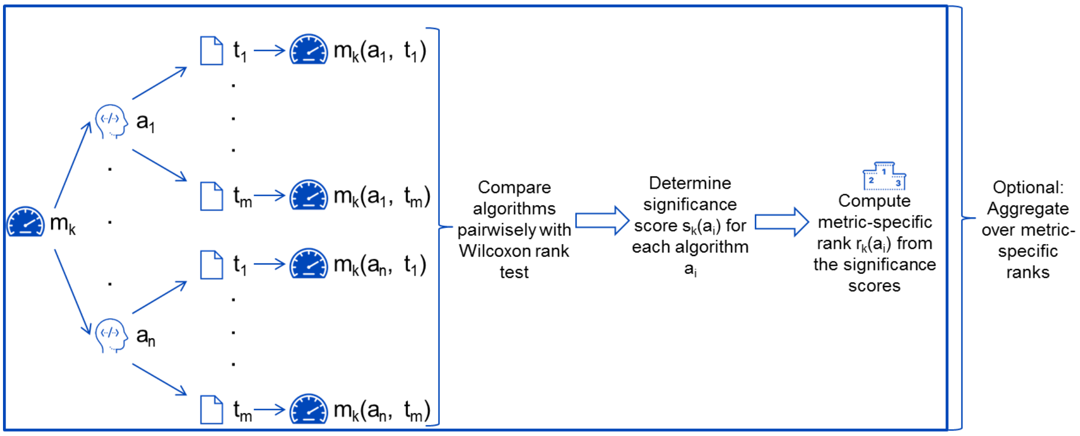

Supplementary Discussion Fig. 3: Significance ranking. Once a ranking has been computed for each metric (as illustrated), the metric ranks can be aggregated. A_i: Algorithm i, i = 1, …, n, t_j: test case j, j = 1, …, m, m_k: metric k, k = 1, …, o. Icons by https://icons8.com/.

Supplementary Notes

Supplementary Note 1

Parameter coverage for different settings

Different challenge platforms

The following challenge venues were considered in the calculation:

- Medical Image Computing and Computer Assisted Intervention (MICCAI): 50% of tasks
- International Symposium on Biomedical Imaging (ISBI): 34% of tasks
- Image Cross Language Evaluation Forum (ImageCLEF): 7% of tasks
- Dialogue on Reverse Engineering Assessments and Methods (DREAM): 3% of tasks
- The International Society for Optical Engineering (SPIE) Medical Imaging: 1% of tasks
- International Conference on Pattern Recognition (ICPR): 1% of tasks
- Kaggle: 1% of tasks
- Challenges with undefined challenge venue: 0.6% of tasks
- Single Molecule Localization Microscopy Symposium (SMLMS): 0.2% of tasks
- American Association of Physicists in Medicine (AAPM): 0.2% of tasks
- International Society for Magnetic Resonance in Medicine (ISMRM): 0.2% of tasks
- Symposium on Statistical Shape Models & Applications (SHAPE Symposium): 0.2% of tasks
- BioImage Informatics (BII): 0.2% of tasks
- Conference at Howard Hughes Medical Institute (HHMI): 0.2% of tasks

As generation of boxplots for a small number of tasks is not meaningful, we combined all the venues that covered less than 1% of all tasks in one joint category (undefined venue, SMLMS, AAPM, ISMRM, SHAPE Symposium, BII, HHMI).

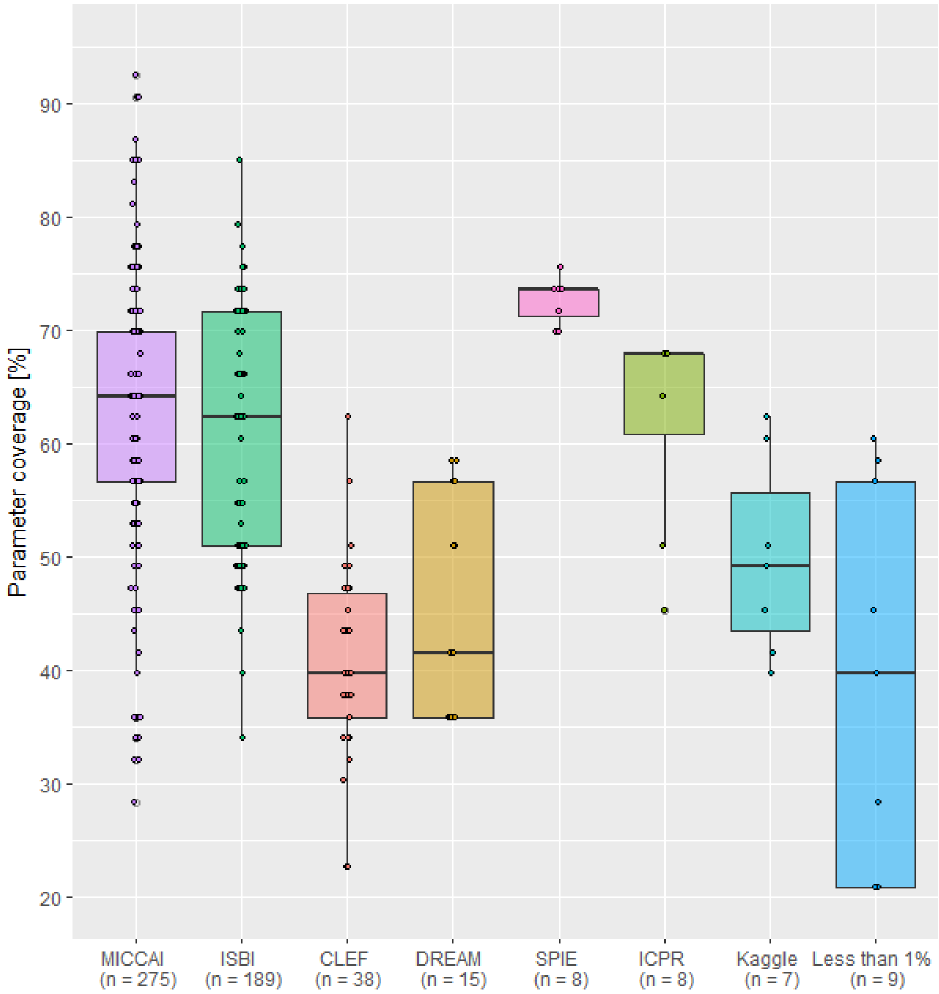

Supplementary Notes Fig. 1: Parameter coverage for different challenge platforms. The center line in the boxplots shows the median, the lower and upper border of the box represent the first and third quartile. The whiskers extend to the lowest value still within 1.5 interquartile range (IQR) of the first quartile, and the highest value still within 1.5 IQR of the third quartile.

Different publication categories

The following challenge venues were considered in the calculation:

- Peer-reviewed journals: 39% of tasks
- Conference proceedings: 36% of tasks
- No report (only website): 25% of tasks

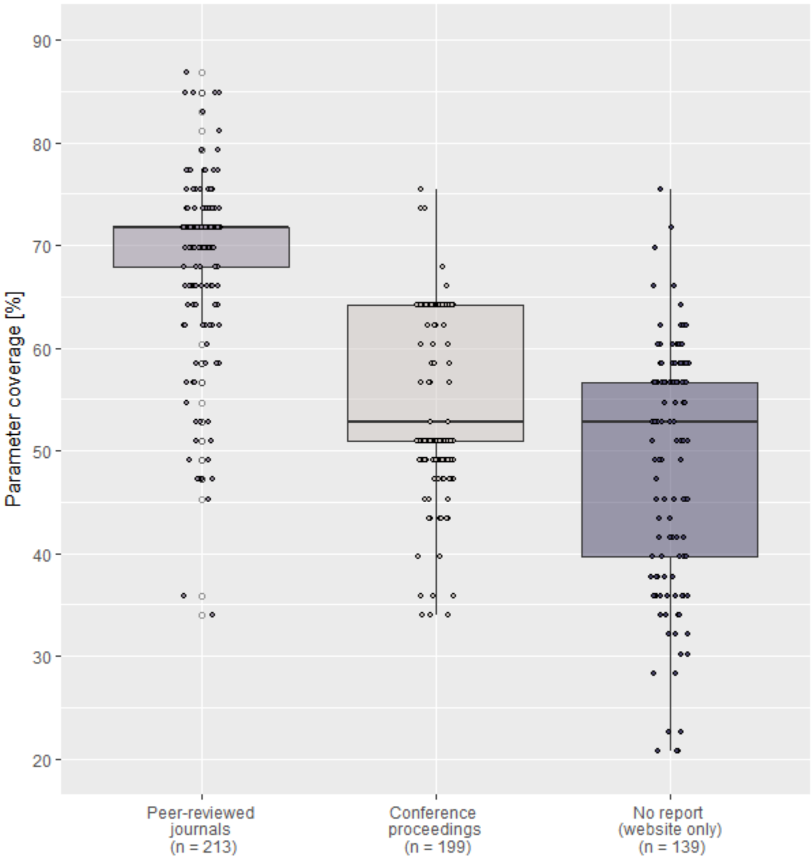

Supplementary Notes Fig. 2: Parameter coverage for different report categories. The center line in the boxplots shows the median, the lower and upper border of the box represent the first and third quartile. The whiskers extend to the lowest value still within 1.5 interquartile range (IQR) of the first quartile, and the highest value still within 1.5 IQR of the third quartile.

Segmentation and other algorithm categories:

For the following calculations, segmentation tasks (70% of tasks) were compared to other algorithm categories (30% of tasks).

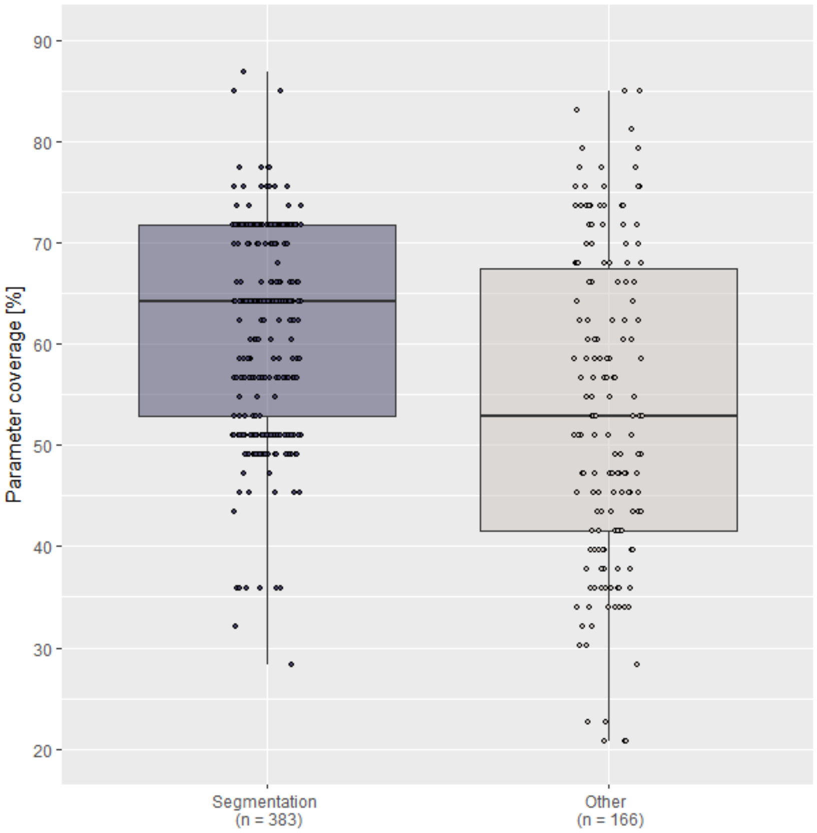

Supplementary Notes Fig. 3: Parameter coverage for segmentation tasks vs. all other algorithm categories. The center line in the boxplots shows the median, the lower and upper border of the box represent the first and third quartile. The whiskers extend to the lowest value still within 1.5 interquartile range (IQR) of the first quartile, and the highest value still within 1.5 IQR of the third quartile.

Different years

For the calculation, we compared challenges hosted

- between 2004 and 2008 (3% of tasks)
- between 2009 and 2012 (18% of tasks)
- between 2013 and 2016 (79% of tasks).

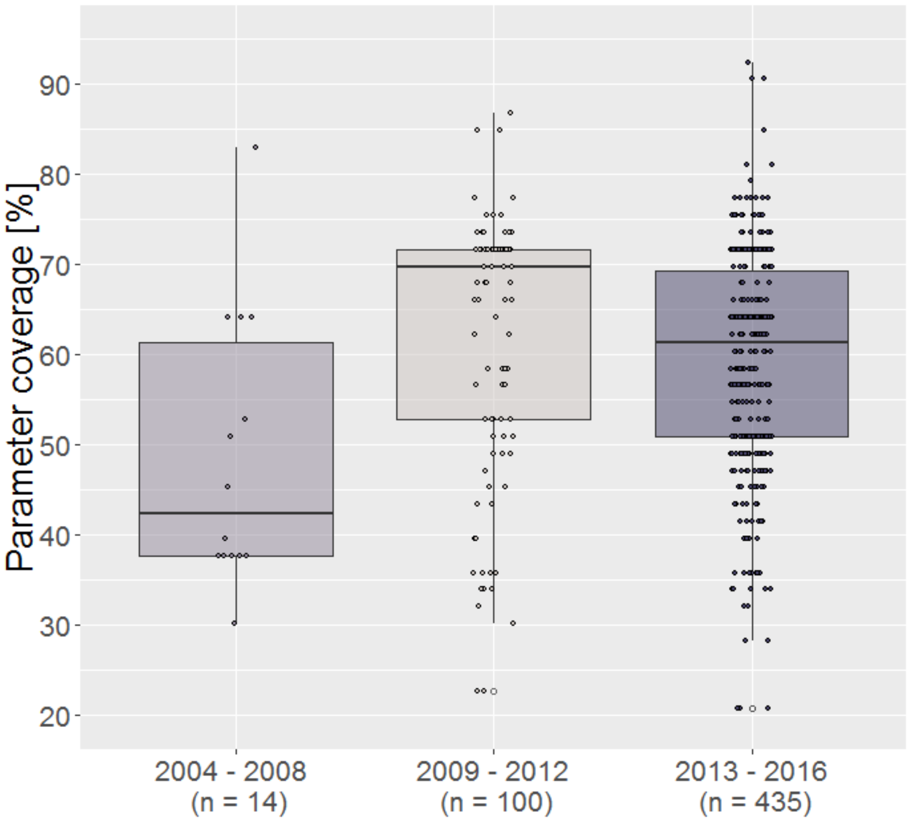

Supplementary Notes Fig. 4: Parameter coverage for different years, grouped in the blocks [2004; 2008], [2009; 2012] and [2013; 2016]. The center line in the boxplots shows the median, the lower and upper border of the box represent the first and third quartile. The whiskers extend to the lowest value still within 1.5 interquartile range (IQR) of the first quartile, and the highest value still within 1.5 IQR of the third quartile.

Supplementary Notes Table 1: List of parameters along with the percentage of challenge tasks for which information on the parameter has been reported

Parameter name	2004 – 2008 [%]	2009 – 2012 [%]	2013 – 2016 [%]
Challenge name*	100	100	100
Challenge website*	100	95	100
Organizing institutions and contact person*	100	91	98
Life cycle type*	100	100	100
Challenge venue or platform	100	100	99
Challenge schedule*	57	43	90
Ethical approval*	0	6	39
Data usage agreement	64	74	57
Interaction level policy*	93	75	58
Organizer participation policy*	0	10	5
Training data policy*	22	24	14
Pre-evaluation method	14	4	5
Evaluation software	21	9	31
Submission format*	50	81	94
Submission instructions	57	78	95
Field(s) of application*	100	98	97
Task category(ies)*	100	99	100
Target cohort*	36	79	62
Algorithm target(s)*	93	99	99
Data origin*	100	96	98
Assessment aim(s)*	14	61	34
Study cohort*	50	85	91
Context information*	14	66	29
Center(s)*	57	61	40
Imaging modality(ies)*	79	97	100
Acquisition device(s)	29	65	16
Acquisition protocol(s)	36	83	71
Operator(s)	29	9	6
Distribution of training and test cases*	33	15	18
Category of training data generation method*	89	87	89
Number of training cases*	89	84	90
Characteristics of training cases*	33	74	80
Annotation policy for training cases*	22	61	29
Annotator(s) of training cases*	89	80	81
Annotation aggregation method(s) for training cases*	22	68	21
Category of test data generation method*	93	72	88

Number of test cases*	93	87	73
Characteristics of test cases*	43	72	79
Annotation policy for test cases*	14	58	29
Annotator(s) of test cases*	64	77	79
Annotation aggregation method(s) for test cases*	43	62	27
Data pre-processing method(s)	14	20	26
Potential sources of reference errors	7	59	22
Metric(s)*	71	93	98
Justification of metrics*	50	21	23
Rank computation method*	21	29	37
Interaction level handling*	43	21	50
Missing data handling*	7	3	22
Uncertainty handling*	7	8	5
Statistical test(s)*	0	12	5
Information on participants	79	90	88
Results	50	81	89
Report document	35	88	72

List of parameters that were identified as relevant when reporting a challenge along with the percentage of challenge tasks for which information on the parameter has been reported (red: < 50%; orange: between 50% and 90%; green: > 90%). Values in percentage are given for different years.

Supplementary Note 2

Results of Questionnaire: Towards next-generation biomedical challenges

For the following analysis, only complete questionnaires (n = 117) and questionnaires with >50% answers (n = 12) were considered. The majority of the participants were professors (30%), PhD students (23%) and postdoctoral researchers (14%) and had a background in engineering, maths, physics or computing (94%). 31% of the participants had already organized a challenge and 63% had taken part in at least one challenge. 92% of all participants agreed that biomedical challenge design should be generally improved. The following problems were identified for the categories data, annotation, evaluation and documentation. For all four categories, we report the most commonly reported problems ordered by frequency of reporting.

Data

Representativeness (33%): Most concerns were related to the representativeness of the data. Criticism was targeted mainly at the representativeness of the training and test sets, balance of the data (e.g. between positives and negatives), selection bias, realism of the data (e.g. with respect to noise and artefacts) and the typically small number of centers/vendors/devices involved. In fact, our analysis revealed that the median number of institutes involved in a challenge was 1 (IQR: (1, 1)) and only 17% of all tasks conducted up to 2016 were based on multi-center data.

One critical consequence of the generally small data sets is that challenge participants tend to complement available training data with their own data sets which makes it impossible to distinguish the effect of an algorithm from the effect of the training data.

Data acquisition (17%): Participants agreed that the data acquisition itself is one of the main barriers for challenge organizers, especially due to legal barriers and high costs. In fact, 22% of challenge organizers encountered problems acquiring the data sets for their challenge. One of the main reasons for participation in a challenge was the access to validation data (30% of challenge participants).

Further problems mentioned include

- Heterogeneity (8%), e.g. due to the lack of acquisition standards
- Infrastructure issues (8%), mainly due to inconsistencies in formats and the lack data management and exploration tools
- Accessibility of the data (8%), mainly after the challenge
- Lack of documentation (8%), especially about the image acquisition process
- Data quality (6%), in general and
- Overfitting/Cheating/Tuning (4%).

Annotation

Quality of reference data (33%): Major concerns were related to errors in the reference data. The annotations were regarded as subjective and/or biased, e.g. because only single observers annotated the data in many cases or automatic tools were used either for the annotations themselves or for the initialization in semi-automatic tools. The lack of quality control in this step was regarded particularly critically for many challenges.

Method for reference generation (16%): A related issue is the method chosen for reference annotation. For example, studies have shown (e.g. [117]) that reference annotations may vary significantly even across medical experts. This issue can potentially be compensated to some extent by merging reference annotations from multiple experts but this was only done (reported) in 73% of all tasks. It should also be noted that 27% of all challenge organizers encountered problems when generating the reference data for their challenge.

Transparency (15%): Lack of transparency was another major issue raised in the context of data annotation. In particular, it was requested to make raw annotations available, report on inter- and intra-observer variability and document how the final reference annotation was generated.

Resources (14%): Another issue raised were the resources required for providing high-quality reference data. The annotation was not only considered particularly challenging but also logistically hard due to improper tools.

Lack of standards (10%): The lack of guidelines for annotating and merging annotations was heavily criticized. Similarly, the lack of standard data formats was regarded critical.

Evaluation

Choice of metric (20%): The metrics applied in current challenges were generally criticized. For example, it was stated that runtime/computational complexity are rarely considered. The metrics are also often not well linked to the clinical context. Finally, optimal metric aggregation is a major issue to be addressed, especially in the case of missing data. On the other hand, participants agreed that finding the right metric(s) for a given task is highly challenging. In fact, 23% of all challenge organizers struggled with the choice of metric(s).

Lack of standards (19%): A major point of criticism was related to the lack of standards with respect to metrics and evaluation frameworks. For example, even the presumably same metrics are sometimes named or applied differently.

Transparency (12%): Missing documentation with respect to the evaluation process was criticized. Concerns were raised regarding the fact that the evaluation criteria are not transparent before the submission of data, potentially allowing organizers to influence the final ranking.

Further difficulties raised include

- Lack of infrastructure/tools (7%) such as public tools for evaluation
- Method for determining ranking (7%), for instance, in the case of missing values
- Lack of quality control (7%), e.g. related to metric implementation
- Too much focus on ranking (5%), especially when considering the sensitivity of the ranks
- Lack of uncertainty handling (4%), especially when considering inter-observer variability as well as
- Lifetime and dynamics (3%) of challenges.

Documentation

Completeness and transparency (47%): Participants agreed that the documentation should be as comprehensive as possible, which is currently not the case. This holds true not only for the reporting of challenge design and results but also for the methods themselves. For example, a challenge ranking should reflect the quality of a method in the context of a given task. Unfortunately, however, method performance may depend crucially on its parameters. Given that only 4% of challenge participants stated that they typically apply their algorithm “as is” to new challenge data, and more than 80% of participants tune their methods to a given task, it comes at a surprise that almost no attention is given to the parameters applied by the algorithms assessed.

Publication of results (13%): Issues with respect to the publication of results further include the delay between submission deadline and paper publication as well as discrepancies between the challenge website and the corresponding publication.

Lifetime and dynamics (10%): Another problem raised was the typically dynamically changing content of challenge websites, which makes proper referencing hard. An open research question is further the optimal lifetime of a challenge considering problems with overfitting, for example.

Further problems related to the documentation were

- Lack of open source code (9%) corresponding to participating algorithms and the evaluation
- Lack of standards for structured reporting (7%), such as common ontologies
- Accessibility of information (5%) especially after the challenge as well as the
- Lack of acknowledgement/citation of all people involved (3%).

Supplementary Note 3

Acknowledgement of all organizers of the 2015 segmentation challenges

We thank all organizers of the 2015 segmentation challenges who are not co-authoring this paper, in particular Cheng-Ta Huang (National Taiwan University of Science and Technology, Taiwan), Chung-Hsing Li (Tri-Service General Hospital, Taiwan), Sheng-Wei Chang (Tri-Service General Hospital, Taiwan), Svitlana Zinger (Eindhoven University of Technology, The Netherlands), Erik Schoon (Catharina Hospital Eindhoven, The Netherlands), Peter de With (Eindhoven University of Technology, The Netherlands), Gustavo Carneiro and Zhi Lu (University of Adelaide, Australia), Jing Wu (Medical University of Vienna, Austria), Ana-Maria Philip (Medical University of Vienna, Austria), Bianca S. Gerendas (Medical University of Vienna, Austria), Sebastian M. Waldstein (Medical University of Vienna, Austria), Ursula Schmidt-Erfurth (Medical University of Vienna, Austria), all involved readers of the OPTIMA team and the VRC (Vienna Reading Center, Austria), Patrik Raudaschl (Institute for Biomedical Image Analysis, UMIT, Austria), Karl Fritscher (Institute for Biomedical Image Analysis, UMIT, Austria), Paolo Zaffino (Magna Graecia University of Catanzaro, Italy), Maria Francesca Spadea (Magna Graecia University of Catanzaro, Italy), Dzung L. Pham (CNRM, The Henry M. Jackson Foundation for the Advancement of Military Medicine, USA), Jerry L. Prince (Johns Hopkins University, USA), Jean-Christophe Houde (Université de Sherbrooke, Canada), Emmanuel Caruyer (CNRS Paris, France), Alessandro Daducci (École Polytechnique Fédérale de Lausanne, Switzerland), Tim Dyrby (Danish Research Centre for Magnetic Resonance, Denmark), Bram Stieltjes (University Hospital Basel, Switzerland), Maxime Descoteaux (Université de Sherbrooke, Canada), Orcun Goksel (ETH Zürich, Switzerland), Antonio Foncubierta-Rodríguez (ETH Zürich, Switzerland), Oscar Alfonso Jiménez del Toro (HES-SO Valais-Wallis, Switzerland), Georg Langs (Medical University of Vienna, Austria), Ivan Eggel (HES-SO Valais-Wallis, Switzerland), Katharina Gruenberg (Radiologisches Zentrum Wiesloch, Germany), Marianne Winterstein (Universitätsklinikum Heidelberg, Germany), Markus Holzer (contextflow GmbH, Austria), Markus Krenn (contextflow GmbH, Austria), Georgios Kontokotsios (EBCONT enterprise technologies GmbH, Austria), Sokratis Metallidis (EBCONT enterprise technologies GmbH, Austria), Roger Schaer (HES-SO Valais-Wallis, Switzerland), András Jakab (Neuroscience Center Zürich, Switzerland), Tomàs Salas Fernandez (Agency for Health Quality and Assessment of Catalonia, Spain), Sebastian Bodenstedt (NCT Dresden, Germany), Martin Wagner (University Hospital Heidelberg, Germany), Hannes Kenngott (University Hospital Heidelberg, Germany), Max Allan (Intuitive Surgical, Inc., USA), Mauricio Reyes (Bern University, Switzerland), Keyvan Farahani (NIH, USA), Jayashree Kalpathy-Cramer (Harvard MGH, USA), Dongjin Kwon (University of Pennsylvania, USA), Heinz Handels (Universität zu Lübeck, Germany), Matthias Liebrand (Universitätsklinikum Schleswig-Holstein, Germany), Ulrike Krämer (Universitätsklinikum Schleswig-Holstein, Germany), Shuo Li (University of Western Ontario, Canada), Stephen M. Damon (Vanderbilt University, USA).

Supplementary Note 4

Acknowledgement of all participants of the international questionnaire

We thank all participants of the international questionnaire, in particular those who filled out > 50% of the form including Daniel Alexander (University College London, UK), Susan Astley (University of Manchester, UK), Peter Bandi (Radboud University Medical Center, The Netherlands), Floris F. Berendsen (Leiden University Medical Center, The Netherlands), Jorge Bernal (Universitat Autonoma de Barcelona, Spain), Zijian Bian (Northeastern University, PRC), Andrew P. Bradley (The University of Queensland, Australia), Paul A. Bromiley (University of Manchester, UK), Esther Bron (Erasmus Medical Center, The Netherlands), Philippe Cattin (University of Basel, Switzerland), Eric Chang (Microsoft Research, PRC), Christos Chatzichristos (National and Kapodistrian University Athens, Greece), Stephane Chauvie (Santa Croce e Carle Hospital, Italy), Xingqiang Chen (Xiamen University, PRC), Veronika Cheplygina (Eindhoven University of Technology, The Netherlands), Guy Cloutier (University of Montreal, Canada), Marleen De Bruijne (Erasmus Medical Center Rotterdam, The Netherlands, and University of Copenhagen, Denmark), Maxime Descoteaux (Université de Sherbrooke, Canada), Fabien Despinoy (LTSI-Inserm U1099, Rennes, France), Thijs Dhollander (The Florey Institute of Neuroscience and Mental Health, Australia), Xiaofei Du (University College London, UK), Sara El Hadji (Politecnico di Milano, Italy), Ahmed El Kaffas (Stanford University, USA), Andrey Fedorov (Brigham and Women's Hospital, USA), Simon Fristed Eskildsen (Aarhus University, Denmark), Babak Ehteshami Bejnordi (Radboud University Medical Center, The Netherlands), Luc Florack (Eindhoven University of Technology, The Netherlands), Yaozong Gao (Apple, USA), Bernard Gibaud (INSERM, Rennes, France), Paul-Gilloteaux (CNRS, Nantes, France), Michael Götz (DKFZ, Germany), Horst Hahn (Fraunhofer MEVIS & Jacobs University Bremen, Germany), Alexander Hammers (King's College London, UK), Yuankai Huo (Vanderbilt University, USA), Allan Hanbury (Technical University Wien, Austria), Luis C. Garcia-Peraza Herrera (University College London, UK), Oscar Jimenez (HES-SO, Switzerland), Leo Joskowicz (The Hebrew University of Jerusalem, Israel), Bernhard Kainz (Imperial College London, UK), Sjoerd Kerkstra (DIAG - Radboud University Medical Center, The Netherlands), Stefan Klein (Erasmus MC, The Netherlands), Michal Kozubek (Masaryk University, Czech Republic), Walter G. Kropatsch (Technical University Wien, Austria), Pablo Lamata (King's College London, UK), Michele Larobina (Italian National Research Council, Italy), Gaby Martins (Instituto Gulbenkian de Ciencia, Portugal), Keno März (DKFZ, Germany), Matthew McCormick (Kitware, Inc., USA), Stephen McKenna (University of Dundee, UK), Karol Miller (UWA, Australia), Erika Molteni (University College London, UK), Cosmin Adrian Morariu (University of Duisburg-Essen, Germany), Henning Müller (HES-SO, Switzerland), Sérgio Pereira (University of Minho, Portugal), Ingerid Reinertsen (SINTEF, Norway), Mauricio Reyes (University of Bern, Switzerland), Constantino Carlos Reyes-Aldasoro (University of London, UK), Gerard Ridgway (University of Oxford, UK), Robert Rohling (UBC, Canada), James Ross (Brigham and Women's Hospital, USA), Danny Ruijters (Philips Healthcare, The Netherlands), Olivier Salvado (CSIRO, Australia), Gerard Sanroma (Universitat Pompeu Fabra, Spain), Ayushi Sinha (Johns Hopkins University, USA), Chetan L. Srinidhi (National Institute of Technology Karnataka, Surathkal, India), Iain Styles (University of Birmingham, UK), Paul Summers (IEO, Italy), Agnieszka Szczotka (University College London, UK), Raphael Sznitman (University of Bern, Switzerland), Sabina Tangaro (Istituto Nazionale di Fisica Nucleare, Italy), Lennart Tautz (Fraunhofer MEVIS, Germany), Sotirios Tsaftaris (The University of Edinburgh, UK), Fons van der Sommen (Eindhoven University of Technology, The Netherlands), Koen Van Leemput (Massachusetts General Hospital, USA), Theo van Walsum (Erasmus Medical Center, The Netherlands), Ching-Wei Wang (National

Taiwan University of Science and Technology, Taiwan), Li Wang (UNC, USA), Michael Wels (Siemens Healthcare GmbH, Germany), Rene Werner (University Medical Center Hamburg-Eppendorf, Germany), Thomas Wollmann (University of Heidelberg, BIOQUANT, IPMB, and DKFZ, Germany), Alistair Young (University of Auckland, New Zealand), Lining Zhang (University College London, UK), Dženan Zukić (Kitware, Inc., USA), Maria A. Zuluaga (Amadeus, France).

Supplementary References

1. Seco, G., de Herrera, A., Schaer, R., Bromuri, S., & Müller, H. Overview of the ImageCLEF 2016 medical task. in *Working Notes of CLEF* (2016).
2. Setio, A. A. A. et al. Validation, comparison, and combination of algorithms for automatic detection of pulmonary nodules in computed tomography images: the LUNA16 challenge. *Med. Image Anal.* **42**, 1-13 (2017).
3. Menze, B. H. et al. Proceedings of MICCAI-BRATS 2016 Multimodal Brain Tumor Image Segmentation Benchmark: “Change Detection”. in *Proc. Workshop Med. Image Anal. Comput. Comput. Assis. Interv.* (2016).
4. Gutman, D. et al. Skin lesion analysis toward melanoma detection: A challenge at the international symposium on biomedical imaging (ISBI) 2016, hosted by the international skin imaging collaboration (ISIC). Preprint at https://arxiv.org/abs/1605.01397. (2016)
5. Karim, R. et al. Segmentation Challenge on the Quantification of Left Atrial Wall Thickness. in *Stat. Atlases Comput. Models Heart* 193-200 (Springer, Cham, 2016).
6. Zheng, G. et al. Evaluation and comparison of 3D intervertebral disc localization and segmentation methods for 3D T2 MR data: A grand challenge. *Med. Image Anal.* **35**, 327-344 (2017).
7. Ulman, V. et al. An objective comparison of cell-tracking algorithms. *Nat. Methods* **14(12)**, 1141 (2017).
8. Wang, C. W. et al. A benchmark for comparison of dental radiography analysis algorithms. *Med. Image Anal.* **31**, 63-76 (2016).
9. De Luca, V. et al. Proceedings of MICCAI 2015 Workshop Challenge on Liver Ultrasound Tracking CLUST 2015. in *Proc. Workshop Med. Image Anal. Comput. Comput. Assis. Interv.* (2015).
10. Wang, C. W. et al. A benchmark for comparison of dental radiography analysis algorithms. *Med. Image Anal.* **31**, 63-76 (2016).
11. Bernal, J. et al. Comparative validation of polyp detection methods in video colonoscopy: results from the MICCAI 2015 Endoscopic Vision Challenge. *IEEE Trans. Med. Imaging* **36(6)**, 1231-1249 (2017).
12. Sirinukunwattana, K. et al. Gland segmentation in colon histology images: The glas challenge contest. *Med. Image Anal.* **35**, 489-502 (2017).
13. Raudaschl, P. F. et al. Evaluation of segmentation methods on head and neck CT: Auto-segmentation challenge 2015. *Med. Phys.* **44(5)**, 2020-2036 (2017).
14. Amin, M. A. & Mohammed, M. K. Overview of the ImageCLEF 2015 Medical Clustering Task. in *Working Notes of CLEF* (2015).
15. Marvasti, N. B., García, M. D. M. R., Üsküdarli, S., Montes, J. F. A., & Acar, B. Overview of the ImageCLEF 2015 liver CT annotation task. in *Working Notes of CLEF* (2015).
16. de Herrera, A. G. S., Müller, H., & Bromuri, S. Overview of the ImageCLEF 2015 Medical Classification Task. in *Working Notes of CLEF* (2015).
17. Maier, O. et al. ISLES 2015-A public evaluation benchmark for ischemic stroke lesion segmentation from multispectral MRI. *Med. Image Anal.* **35**, 250-269 (2017).
18. Maier-Hein, K. H. et al. The challenge of mapping the human connectome based on diffusion tractography. *Nat. commun.* **8(1)**, 1349 (2017).
19. Maier-Hein, K. H. et al. Tractography-based connectomes are dominated by false-positive connections. Preprint at https://www.biorxiv.org/content/early/2016/11/07/084137 (2016).
20. Suinesiaputra, A. et al. Statistical shape modeling of the left ventricle: myocardial infarct classification challenge. *IEEE J. Biomed. Health Inform*. 22(2), 503-515 (2017).
21. Carass, A. et al. Longitudinal multiple sclerosis lesion segmentation: resource and challenge. *NeuroImage* **148**, 77-102 (2017).
22. Armato, S. G. et al. LUNGx Challenge for computerized lung nodule classification. *J. Med. Imaging* **3(4)**, 044506 (2016).
23. Menze, B. H. et al. Proceedings of Multimodal Brain Tumor Image Segmentation Challenge held in conjunction with MICCAI 2015. in *Proc. Workshop Med. Image Anal. Comput. Comput. Assis. Interv.* (2015).
24. Wu, J. et al. Multivendor spectral-domain optical coherence tomography dataset, observer annotation performance evaluation, and standardized evaluation framework for intraretinal cystoid fluid segmentation. *J. Ophthalmol.* (2016).

25. Goksel, O. et al. Overview of the VISCERAL Challenge at ISBI 2015. in *Proc. IEEE Int. Symp. Biomed. Imaging* 6-11 (IEEE, 2015).
26. Ferizi, U. et al. Diffusion MRI microstructure models with in vivo human brain Connectome data: results from a multi-group comparison. *NMR Biomed.* **30(9)**, e3734 (2017).
27. Wang, C. W. et al. Evaluation and comparison of anatomical landmark detection methods for cephalometric x-ray images: A grand challenge. *IEEE Trans. Med. Imaging* **34(9)**, 1890-1900 (2015).
28. Bernard, O. et al. Standardized evaluation system for left ventricular segmentation algorithms in 3D echocardiography. *IEEE Trans. Med. Imaging* **35(4)**, 967-977 (2016).
29. De Luca, V. et al. The 2014 liver ultrasound tracking benchmark. *Phys. Med. Biol.* **60(14)**, 5571 (2015).
30. Yao, J., & Li, S. Report of vertebra segmentation challenge in 2014 MICCAI Workshop on computational spine imaging. in *Recent Advances in Computational Methods and Clinical Applications for Spine Imaging* 247-259 (Springer, Cham, 2015).
31. Bron, E. E. et al. Standardized evaluation of algorithms for computer-aided diagnosis of dementia based on structural MRI: the CADDementia challenge. *NeuroImage* **111**, 562-579 (2015).
32. IPAL Lab. MITOS & ATYPIA. Detection of mitosis and evaluation of nuclear atypia score in breast cancer histological images. (2014) at http://ludo17.free.fr/mitos_atypia_2014/icpr2014_MitosAtypia_DataDescription.pdf
33. nar Yolum, P., Üsküdarl, S., & Acar, B. ImageCLEF Liver CT Image Annotation Task 2014. in *CLEF Working Notes* (2014).
34. Wolterink, J. M. et al. An evaluation of automatic coronary artery calcium scoring methods with cardiac CT using the orCaScore framework. *Med. Phys.* **43(5)**, 2361-2373 (2016).
35. Pujol, S. et al. The DTI challenge: toward standardized evaluation of diffusion tensor imaging tractography for neurosurgery. *J Neuroimaging* **25(6)**, 875-882 (2015).
36. Menze, B. H. et al. The multimodal brain tumor image segmentation benchmark (BRATS). *IEEE Trans. Med. Imaging* **34(10)**, 1993 (2015).
37. Lu, Z. et al. Evaluation of three algorithms for the segmentation of overlapping cervical cells. *IEEE journal of biomedical and health informatics* **21(2)**, 441-450 (2017).
38. Jimenez-del-Toro, O. et al. Cloud-based evaluation of anatomical structure segmentation and landmark detection algorithms: VISCERAL anatomy benchmarks. *IEEE Trans. Med. Imaging* **35(11)**, 2459-2475 (2016).
39. Veta, M. et al. Assessment of algorithms for mitosis detection in breast cancer histopathology images. *Med. Image Anal.* **20(1)**, 237-248 (2015).
40. Maška, M. et al. A benchmark for comparison of cell tracking algorithms. *Bioinform.* **30(11)**, 1609-1617 (2014).
41. Hogeweg, L. et al. Clavicle segmentation in chest radiographs. *Med. Image Anal.* **16(8)**, 1490-1502 (2012).
42. de Herrera, A. G. S., Kalpathy-Cramer, J., Demner-Fushman, D., Antani, S. K., & Müller, H. Overview of the ImageCLEF 2013 Medical Tasks. in *CLEF Working Notes* (2013).
43. Tobon-Gomez, C. et al. Benchmark for algorithms segmenting the left atrium from 3D CT and MRI datasets. *IEEE Trans. Med. Imaging* **34(7)**, 1460-1473 (2015).
44. Mendrik, A. M. et al. MRBrainS challenge: online evaluation framework for brain image segmentation in 3T MRI scans. *Comput. Intell. Neurosci.*, 1 (2015).
45. Sage, D. et al. Quantitative evaluation of software packages for single-molecule localization microscopy. *Nat. Methods* **12(8)**, 717 (2015).
46. Langs, G., Aziz Taha, A., Menze, B., Hanbury, A. Visceral. Definition of the evaluation protocol and goals for Competition 1. (2013) at http://www.visceral.eu/assets/Uploads/Deliverables/VISCERAL-D4.1.pdf
47. Rueda, S. et al. Evaluation and comparison of current fetal ultrasound image segmentation methods for biometric measurements: a grand challenge. *IEEE Trans. Med. Imaging* **33(4)**, 797-813 (2014).
48. Karim, R. et al. Evaluation of state-of-the-art segmentation algorithms for left ventricle infarct from late Gadolinium enhancement MR images. *Med. Image Anal.* **30**, 95-107 (2016).
49. Kirişli, H. A. et al. Standardized evaluation framework for evaluating coronary artery stenosis detection, stenosis quantification and lumen segmentation algorithms in computed tomography angiography. *Med. Image Anal.* **17(8)**, 859-876 (2013).

50. Küffner, R. et al. Crowdsourced analysis of clinical trial data to predict amyotrophic lateral sclerosis progression. *Nat. Biotechnol.* **33(1)**, 51 (2015).
51. Landman, B., & Warfield, S. MICCAI 2012 workshop on multi-atlas labeling. in *Proc. Med. Image Comput. Comput. Assist. Interv.* (Springer, 2012).
52. Daducci, A. et al. Quantitative comparison of reconstruction methods for intra-voxel fiber recovery from diffusion MRI. *IEEE Trans. Med. Imaging* **33**, 384-399 (2014).
53. Müller, H. et al. Overview of the ImageCLEF 2012 Medical Image Retrieval and Classification Tasks. In in *Working Notes of CLEF* 1-16(CLEF, 2012).
54. Aptoula, E., Courty, N., & Lefevre, S. Mitosis detection in breast cancer histological images with mathematical morphology. in *Signal Process. Commun. Appl.* 1-4 (IEEE, 2013).
55. Išgum, I. et al. Evaluation of automatic neonatal brain segmentation algorithms: the NeoBrainS12 challenge. *Med. Image Anal.* **20(1)**, 135-151 (2015).
56. Wang, L. et al. MICCAI 2012 Workshop on Novel Imaging Biomarkers for Alzheimer's Disease and Related Disorders. in *Proc. Workshop Med. Image Anal. Comput. Comput. Assis. Interv.* (2016).
57. Chenouard, N. et al. Objective comparison of particle tracking methods. *Nat. Methods* **11(3)**, 281 (2014).
58. Foggia, P., Percannella, G., Soda, P., & Vento, M. Benchmarking HEp-2 cells classification methods. *IEEE Trans. Med. Imaging* **32(10)**, 1878-1889 (2013).
59. Litjens, G. et al. Evaluation of prostate segmentation algorithms for MRI: the PROMISE12 challenge. *Med. Image Anal.* **18(2)**, 359-373 (2014).
60. Petitjean, C. et al. Right ventricle segmentation from cardiac MRI: a collation study. *Med. Image Anal.* **19(1)**, 187-202 (2015).
61. Arganda-Carreras, I. et al. Crowdsourcing the creation of image segmentation algorithms for connectomics. *Front. Neuroanat.* **9**, 142 (2015).
62. Rudyanto, R. D. et al. Comparing algorithms for automated vessel segmentation in computed tomography scans of the lung: the VESSEL12 study. *Med. Image Anal.* **18(7)**, 1217-1232 (2014).
63. Kalpathy-Cramer, J., Müller, H., Bedrick, S., Eggel, I., de Herrera, A. G. S., & Tsikrika, T. Overview of the CLEF 2011 Medical Image Classification and Retrieval Tasks. in *Working Notes of CLEF* 97-112 (2011).
64. Balocco, S. et al. Standardized evaluation methodology and reference database for evaluating IVUS image segmentation. *Comput. Med. Imaging Graph.* **38(2)**, 70-90 (2014).
65. Tobon-Gomez, C. et al. Benchmarking framework for myocardial tracking and deformation algorithms: An open access database. *Med. Image Anal.* **17(6)**, 632-648 (2013).
66. Suinesiaputra, A. et al. A collaborative resource to build consensus for automated left ventricular segmentation of cardiac MR images. *Med. Image Anal.* **18(1)**, 50-62 (2014).
67. Pop, M. et al. EP challenge-STACOM'11: forward approaches to computational electrophysiology using MRI-based models and in-vivo CARTO mapping in swine hearts. in *Stat. Atlases Comput. Models Heart* 1-13 (Springer, Berlin, Heidelberg, 2011).
68. Liu, Yuan. The DIADEM and beyond. *Neuroinform.*, 99-102 (2011).
69. Murphy, K. et al. Evaluation of registration methods on thoracic CT: the EMPIRE10 challenge. *IEEE Trans. Med. Imaging* **30(11)**, 1901-1920 (2011).
70. Yang, X. et al. Automated segmentation of the parotid gland based on atlas registration and machine learning: a longitudinal MRI study in head-and-neck radiation therapy. *Int. J. Radiat. Oncol. Biol. Phys.* **90(5)**, 1225-1233 (2014).
71. Pekar, V., Allaire, S., Qazi, A. A., Kim, J. J., & Jaffray, D. A. Head and neck auto-segmentation challenge: segmentation of the parotid glands. in *Proc. Med. Image Comput. Comput. Assis. Interv.* (2010).
72. Gurcan, M. N., Madabhushi, A., & Rajpoot, N. Pattern recognition in histopathological images: An ICPR 2010 contest. in *Recognit. Pattern Signals Speech Images. Videos* 226-234 (Springer, Berlin, Heidelberg, 2010).
73. Heimann, T. et al. Segmentation of knee images: a grand challenge. in *Proc. Workshop Med. Image Anal. Clinic* 207-214(Springer, 2010).
74. Van Ginneken, B. et al. Comparing and combining algorithms for computer-aided detection of pulmonary nodules in computed tomography scans: the ANODE09 study. *Med. Image Anal.* **14(6)**, 707-722 (2010).
75. Lo, P. et al. Extraction of airways from CT (EXACT'09). *IEEE Trans. Med. Imaging* **31(11)**, 2093-2107 (2012).
76. Pekar, V., Allaire, S., Kim, J., & Jaffray, D. A. Head and neck auto-segmentation challenge. *MIDAS J.* **11** (2009).

77. Niemeijer, M. et al. Retinopathy online challenge: automatic detection of microaneurysms in digital color fundus photographs. *IEEE Trans. Med. Imaging* **29(1)**, 185-195 (2010).
78. Hameeteman, K. et al. Evaluation framework for carotid bifurcation lumen segmentation and stenosis grading. *Med. Image Anal.* **15(4)**, 477-488 (2011).
79. Brown, M. et al. The Second International Workshop on Pulmonary Image Analysis. in *Proc. Med. Image Comput. Comput. Assist. Interv.* (CreateSpace Independent Publishing Platform, 2009).
80. Schaap, M. et al. Standardized evaluation methodology and reference database for evaluating coronary artery centerline extraction algorithms. *Med. Image Anal.* 13(5), 701-714 (2009).
81. Styner, M. et al. 3D segmentation in the clinic: A grand challenge II: MS lesion segmentation. *MIDAS J.*, 1-6 (2008).
82. Deselaers, T., Deserno, T. M., & Müller, H. Automatic medical image annotation in ImageCLEF 2007: Overview, results, and discussion. *Pattern Recognit. Lett.* 29(15), 1988-1995 (2008).
83. Heimann, T. et al. Comparison and evaluation of methods for liver segmentation from CT datasets. *IEEE Trans. Med. Imaging* **28(8)**, 1251-1265 (2009).
84. Munafò, M. R. et al. A manifesto for reproducible science. Nat. Hum. Behav. 1, 0021 (2017).
85. Jannin, P., Grova, C. & Maurer, C. R. Model for defining and reporting reference-based validation protocols in medical image processing. Int. J. CARS **1(2)**, 63-73 (2006).
86. Zendel, O., Murschitz, M., Humenberger, M. & Herzner, W. How good is my test data? Introducing safety analysis for computer vision. Int. J. Comput. Vis. **125**, 95-109 (2017).
87. Jannin, P. & Korb, W. *Assessment of image-guided interventions.* in Image-Guided Interventions 531-549 (Springer, Boston, MA, 2008).
88. Haynes, B. & Haines, A. Barriers and bridges to evidence based clinical practice. *Bmj* **317(7153)**, 273-276 (1998).
89. Ioannidis, J. P. Why most clinical research is not useful. *PLoS Med.* **13(6)**, e1002049 (2016).
90. Norman, D. A. & Verganti, R. Incremental and radical innovation: Design research vs. technology and meaning change. *Design issues* **30(1)**, 78-96.
91. Cutler, D.M. & McClellan, M. Is technological change in medicine worth it? *Health affairs* **20(5)**, 11-29 (2001).
92. Shekelle, P., Morton, S. C. & Keeler, E. B. Costs and benefits of health information technology. Evid. *Rep. Technol. Assess.* **132**, 1-71 (2006).
93. Black, A. D. et al. The impact of eHealth on the quality and safety of health care: a systematic overview. *PLoS Med.* **8(1)**, e1000387 (2011).
94. Garg, A. X. et al. Effects of computerized clinical decision support systems on practitioner performance and patient outcomes: a systematic review. *Jama* **293(10)**, 1223-1238 (2005).
95. Barnes, D., Wilkerson, T., & Stephan, M. Contributing to the development of grand challenges in maths education. in *Proc. Int. Congress on Math. Educ.* 703-704 (Springer, Cham, 2017).
96. Peng, H. et al. BigNeuron: large-scale 3D neuron reconstruction from optical microscopy images. *Neuron* **87(2)**, 252-256 (2015).
97. Masko, D. & Hensman, P. The impact of imbalanced training data for convolutional neural networks. *Bachelor thesis, KTH, School of Computer Science and Communication* (2015).
98. Jain, A. K. & Chandrasekaran, K. Dimensionality and sample size considerations in pattern recognition practice. in *Handbook of Statistics* **2**, 835-855 (Elsevier, 1982).
99. Raudys, S. & Jain, A. Small sample size effects in statistical pattern recognition: recommendations for practitioners. *IEEE Trans. Pattern Anal. Mach. Intell.* **3**, 252-264 (1991).
100. Kalayeh, H. M. & Landgrebe, D. A. Predicting the required number of training samples. *IEEE Trans. Pattern Anal. Mach. Intell.* **5(6)**, 664-667 (1983).
101. Bonnett, D. G. Sample size requirements for estimating intraclass correlations with desired precision. *Stat. Med.* **21(9)**, 1331-1335 (2002).
102. Shoukri, M. M., Asyali, M. H. & Donner, A. Sample size requirements for the design of reliability study: review and new results. *Stat. Methods. Med. Res.* **13(4)**, 251-271 (2004)
103. Sim, J. & Wright, C. C. The kappa statistic in reliability studies: use, interpretation, and sample size requirements. *Physical. Ther.* **85(3)**, 257-268 (2005)
104. Deng, J. et al. ImageNet: a large-scale hierarchical image database. in *Proc. IEEE Conf. Comp. Vis. Pattern Recognit.* 248-255 (IEEE, 2009).
105. Everingham, M., Gool, L. V., Williams, C. K. I., Winn, J. & Zisserman, A. The pascal visual object classes (VOC) challenge. *Int. J. Comput. Vis.* **88**, 303-338 (2010).
106. Chum, O., Philbin, J., Isard, M. & Zisserman, A. Scalable near identical image and shot detection. in *Proc. ACM Conf. Image Video Retrieval* 549-556 (ACM, 2007).
107. Torralba, A. & Alexei A. E. Unbiased look at dataset bias. in *Proc. IEEE Conf. Comp. Vis. Pattern Recognit.* 1521-1528. (IEEE, 2011).
108. Grünberg, K. et al. Annotating Medical Image Data. in *Cloud-Based Benchmarking of Med. Image Anal.* 45-67 (Springer, Cham, 2017).
109. Bamberg, F. et al. Whole-body MR imaging in the german national cohort: rationale, design, and technical background. *Radiol.* **277(1)**, 206-220 (2015).

110. Schlett, C. L. et al. Population-based imaging and radiomics: rationale and perspective of the german national cohort MRI study. *RöFo* **188(7)**, 652-661 (2016).
111. Melsaether, A. et al. Inter-and intrareader agreement for categorization of background parenchymal enhancement at baseline and after training. *AJR Am. J. Roentgenol.* **203(1)**, 209-215 (2014).
112. Welinder, P. & Perona, P. Online crowdsourcing: rating annotators and obtaining cost-effective labels. in *Proc. IEEE Comput. Soc. Conf. Comput. Vis. Pattern Recognit.* 25-32 (IEEE, 2010).
113. Bartlett, J. W. & Frost, C. Reliability, repeatability and reproducibility: analysis of measurement errors in continuous variables. *Ultrasound Obstet. Gynecol.* **31(4)**, 466-475 (2008).
114. Van Den Heuvel, E. R. & Trip, A. Evaluation of measurement systems with a small number of observers. *Quality Engineering* **15(2)**, 323-331 (2002).
115. Walter, S. D., Eliasziw, M. & Donner, A. Sample size and optimal designs for reliability studies. *Stat. Med.* **17(1)**, 101-110 (1998).
116. Asman, A. J. & Landman, B. A. Robust statistical label fusion through consensus level, labeler accuracy, and truth estimation (COLLATE). *IEEE Trans. Med. Imaging* **30(10)**, 1779-1794 (2011).
117. Lampert, T. A., Stumpf, A. & Gancarski, P. An empirical study into annotator agreement, ground truth estimation, and algorithm evaluation. *IEEE Trans. Image Process.* **25(6)**, 2557-2572 (2016).
118. Sheng, V. S., Provost, F., & Ipeirotis, P. G. Get another label? Improving data quality and data mining using multiple, noisy labelers. in *Proc. ACM Int. Conf. KDD* 614-622 (ACM, 2008).
119. Tian, T. & Zhu, J. Max-margin majority voting for learning from crowds. in *Adv. Neural Inf. Process. Syst.* 1621-1629 (2015).
120. Warfield, S. K., Zou, K. H. & Wells, W. M. Simultaneous truth and performance level estimation (STAPLE): an algorithm for the validation of image segmentation. *IEEE Trans. Med. Imaging* **23**, 903-921 (2004).
121. Long, C., Hua, G. & Kapoor, A. Active visual recognition with expertise estimation in crowdsourcing. in *Proc. IEEE Int. Conf. Comput. Vis.* 3000-3007 (IEEE, 2013).
122. Peng, B. & Zhang, L. Evaluation of image segmentation quality by adaptive ground truth composition. in *Comput. Vis. ECCV* 287-300 (Springer, Berlin, Heidelberg, 2012).
123. Von Ahn, L. & Dabbish, L. Labeling images with a computer game. in *Proc. SIGCHI Conf. Hum. Factor. Comput. Syst.* 319-326 (ACM, 2004).
124. Branson, S., Van Horn, G. & Perona, P. Lean crowdsourcing: combining humans and machines in an online system. in *Proc. IEEE Conf. Comput. Vis. Pattern Recognit.* 7474-7483 (IEEE, 2017).
125. Taha, A. A. & Hanbury, A. Metrics for evaluating 3D medical image segmentation: analysis, selection, and tool. *BMC Med. Imaging* **15(1)**, 29 (2015).
126. Ulman, V. et al. An objective comparison of cell-tracking algorithms. *Nat. Meth* **14(12)**, 1141 (2017).
127. Taha, A. A., Hanbury, A. & Jiménez-del-Toro, O. A. A formal method for selecting evaluation metrics for image segmentation. in *Proc. IEEE Int. Conf. Image Proc.* 932-936 (IEEE, 2014).
128. Pont-Tuset, J. & Marques, F. Measures and meta-measures for the supervised evaluation of image segmentation. in *Proc. IEEE Conf. Comput. Vis. Pattern Recognit.* 2131-2138. (IEEE, 2013).
129. Cehovin, L., Kristan, M. & Leonardis, A. Is my new tracker really better than yours? in *IEEE Winter Conf. Appl. Comput. Vis.* 540-547 (IEEE, 2014).
130. Chenouard, N. et al. Objective comparison of particle tracking methods. *Nat. Meth.* **11**, 281-289 (2014).
131. Menze, B. H. et al. The multimodal brain tumor image segmentation benchmark (BRATS). *IEEE Trans. Med. Imaging* **34**, 1993-2024 (2015).
132. Johannsen, O. et al. A taxonomy and evaluation of dense light field depth estimation algorithms. in *Proc. IEEE Conf. Comput. Vis. Pattern Recognit. Workshops* 82-99 (IEEE, 2017).
133. Honauer, K., Johannsen, O., Kondermann, D. & Goldluecke, B. A dataset and evaluation methodology for depth estimation on 4d light fields. in *Asian Conf. Comput. Vis.* 19-34 (Springer, Cham, 2016).
134. Honauer, K., Maier-Hein, L. & Kondermann, D. The HCI stereo metrics: geometry-aware performance analysis of stereo algorithms. in *Proc. IEEE Int. Conf. Comput. Vis.* 2120-2128 (IEEE, 2015).
135. Langville, A. N. & Carl D. Meyer. *Who's #1? The Science of Rating and Ranking.* (Princeton University Press, 2012).
136. Pang, Y. & Ling, H. Finding the best from the second bests-inhibiting subjective bias in evaluation of visual tracking algorithms. in *Proc. IEEE Int. Conf. Comput. Vis.* 2784-2791. (IEEE, 2013).
137. Kristan, M. et al. A novel performance evaluation methodology for single-target trackers. *IEEE Trans. Pattern Anal. Mach. Intell.* **38(11)**, 2137-2155 (2016).
138. Cabezas, I., Trujillo, M. & Florian, M. A non-linear quantitative evaluation approach for disparity estimation - pareto dominance applied in stereo vision. in *Proc. Int. Joint Conf. Comput. Vis. Computer. Graph. Theory Appl.* 704-709. (2011).
139. Neilson, D. & Yang, Y. H. Evaluation of constructable match cost measures for stereo correspondence using cluster ranking. in *Proc. IEEE Conf. Comput. Vis. Pattern Recognit.* 1-8. (IEEE, 2008).

140. Taha, A. A. & Hanbury, A. Evaluation metrics for medical organ segmentation and lesion detection. in *Cloud-Based Benchmarking of Medical Image Analysis* 87-105 (Springer International Publishing, 2017).
141. Hanbury, A., Müller, H. & Langs, G. *Cloud-Based Benchmarking of Medical Image Analysis.* (Springer International Publishing, 2017).
142. Fei-Fei, L., Fergus, R. & Perona, P. One-shot learning of object categories. *IEEE Trans. Pattern Anal. Mach. Intell.* **28(4)**, 594-611 (2006).
143. Perazzi, F. et al. A benchmark dataset and evaluation methodology for video object segmentation. in *Proc. IEEE Conf. Comput. Vis. Pattern Recognit.* 724-732 (IEEE, 2016).
144. Dwork, C., et al. Preserving statistical validity in adaptive data analysis. in *Proc. ACM Symp. Theory Comput.* 117-126 (ACM, 2015).
145. Dream Challenges. DREAM Challenges. (2006) at http://dreamchallenges.org/ (Accessed: 16th July 2018)
146. Kaggle Inc. The Home of Data Science & Machine Learning. (2010) at https://www.kaggle.com/ (accessed: 20th February 2018)
147. Microsoft Azure. CodaLab - Accelerating reproducible computational research. (2014) at https://competitions.codalab.org/ (accessed: 20th February 2018)
148. Consortium for Open Medical Image Computing. Grand Challenges in Biomedical Image Analysis. (2012) at https://grand-challenge.org/ (accessed: 20th February 2018)
149. Kitware. COVALIC. at https://challenge.kitware.com/#challenges/learn (accessed: 15th May 2017)
150. crowdAI Inc. Solve real-world problems using open data. (2016) at http://crowdai.org/ (accessed: 20th February 2018)
