# Supplementary material for: Why rankings of biomedical image analysis competitions should be interpreted with care
Source: Nat Commun. 2018 Dec 6;9:5217. doi: 10.1038/s41467-018-07619-7 (PMC6284017; doi:10.1038/s41467-018-07619-7)
Supplement: Supplementary file 3 — Reporting Summary [file 41467_2018_7619_MOESM3_ESM.pdf]

nature research

Corresponding author(s): Lena Maier-Hein

Reporting Summary

Nature Research wishes to improve the reproducibility of the work that we publish. This form provides structure for consistency and transparency in reporting. For further information on Nature Research policies, see Authors & Referees and the Editorial Policy Checklist.

Statistical parameters

When statistical analyses are reported, confirm that the following items are present in the relevant location (e.g. figure legend, table legend, main text, or Methods section).

n/a	Confirmed	
☐	☒	The exact sample size (*n*) for each experimental group/condition, given as a discrete number and unit of measurement
☒	☐	An indication of whether measurements were taken from distinct samples or whether the same sample was measured repeatedly
☒	☐	The statistical test(s) used AND whether they are one- or two-sided<br>*Only common tests should be described solely by name; describe more complex techniques in the Methods section.*
☒	☐	A description of all covariates tested
☒	☐	A description of any assumptions or corrections, such as tests of normality and adjustment for multiple comparisons
☒	☐	A full description of the statistics including central tendency (e.g. means) or other basic estimates (e.g. regression coefficient) AND variation (e.g. standard deviation) or associated estimates of uncertainty (e.g. confidence intervals)
☒	☐	For null hypothesis testing, the test statistic (e.g. *F*, *t*, *r*) with confidence intervals, effect sizes, degrees of freedom and *P* value noted<br>*Give P values as exact values whenever suitable.*
☒	☐	For Bayesian analysis, information on the choice of priors and Markov chain Monte Carlo settings
☒	☐	For hierarchical and complex designs, identification of the appropriate level for tests and full reporting of outcomes
☒	☐	Estimates of effect sizes (e.g. Cohen's *d*, Pearson's *r*), indicating how they were calculated
☐	☒	Clearly defined error bars<br>*State explicitly what error bars represent (e.g. SD, SE, CI)*

Our web collection on statistics for biologists may be useful.

Software and code

Policy information about availability of computer code

Data collection	Collected data sets<br>- DS1: Captured biomedical challenges from publicly available sources (2004 to 2016)<br>- DS2: 2015 segmentation challenges results provided by challenge organizers<br>- DS3: Individual (but anonymized) responses to survey “Towards next-generation biomedical challenges”<br>- DS4: Individual (but anonymized) responses to survey regarding refinement of parameter list<br><br>A graphical tool for capturing biomedical challenges was developed. The tool is based on an Eclipse Modeling Framework (EMF) Ecore meta model. Java code of the model and the user interface is generated automatically based on that meta model. EMF Forms is used as UI technology. Eclipse Modeling Tools (Version: Neon.2 Release (4.6.2)) was used as integrated development environment (IDE), which is a dedicated variant of the Eclipse IDE used for model-driven software development based on the EMF. The tool can be executed within the IDE. Built-in export functionality to XMI format is provided (resulting data set: DS1). Custom Java code was developed to export captured challenge data to Microsoft Excel and CSV for further processing by using Apache POI 3.14 (Java API for Microsoft documents).<br><br>The web tool eSurvey Creator (https://www.esurveycreator.com/) was used to create the questionnaires, conduct the surveys, collect all responses and export the collected results to Microsoft Excel or PDF as data sets DS3 and DS4.
Data analysis	DS1 was analyzed w.r.t. comprehensive reporting. Custom Java code was developed to perform the descriptive statistical analysis described in Methods, section “Experiment: Comprehensive reporting”.

DS2 was analyzed w.r.t. robustness of rankings. Custom R code was developed for the experiment described in Methods, section "Experiment: Sensitivity of challenge ranking". The statistical analyses were performed with R version 3.4.3 (The R Foundation for Statistical Computing 2017).

The web tool eSurvey Creator (https://www.esurveycreator.com/) was used to analyze DS3 and DS4 and aggregated reports provided by this tool. Manually aggregated results from DS3 can be found in Supplementary Notes 2. Results from DS4 which were automatically evaluated by eSurvey Creator were incorporated into the parameter list (Supplementary Table 2).

For manuscripts utilizing custom algorithms or software that are central to the research but not yet described in published literature, software must be made available to editors/reviewers upon request. We strongly encourage code deposition in a community repository (e.g. GitHub). See the Nature Research guidelines for submitting code & software for further information.

Data

Policy information about availability of data

All manuscripts must include a data availability statement. This statement should provide the following information, where applicable:

- Accession codes, unique identifiers, or web links for publicly available datasets
- A list of figures that have associated raw data
- A description of any restrictions on data availability

Four data sets were generated and analyzed during the current study:
- DS1: Captured biomedical challenges from publicly available sources (2004 to 2016)
- DS2: 2015 segmentation challenges results provided by challenge organizers
- DS3: Individual responses to survey "Towards next-generation biomedical challenges"
- DS4: Individual responses to survey regarding refinement of parameter list

DS1 is available from http://doi.org/10.5281/zenodo.1453313. DS2 is not publicly available as it contains information that could compromise challenge participants' privacy or consent. DS3 and DS4 are available from the corresponding author L.M.-H. upon reasonable request.

Field-specific reporting

Please select the best fit for your research. If you are not sure, read the appropriate sections before making your selection.

☒ Life sciences ☐ Behavioural & social sciences ☐ Ecological, evolutionary & environmental sciences

For a reference copy of the document with all sections, see nature.com/authors/policies/ReportingSummary-flat.pdf

Life sciences study design

All studies must disclose on these points even when the disclosure is negative.

Sample size

DS1 - See Methods (Inclusion criteria for "Experiment: Comprehensive reporting"):
Our aim was to capture all biomedical image analysis challenges that have been conducted up to 2016, hence, no sample size calculation was necessary. We did not include 2017 challenges as our focus is on information provided in scientific papers, which may have a delay of more than a year to be published after challenge execution. To acquire the data, we analyzed the websites hosting/representing biomedical image analysis challenges, namely https://grand-challenge.org, dreamchallenges.org and kaggle.com as well as websites of main conferences in the field of biomedical image analysis, namely Medical Image Computing and Computer Assisted Intervention (MICCAI), International Symposium on Biomedical Imaging (ISBI), International Society for Optics and Photonics (SPIE) Medical Imaging, Cross Language Evaluation Forum (CLEF), International Conference on Pattern Recognition (ICPR), The American Association of Physicists in Medicine (AAPM), the Single Molecule Localization Microscopy Symposium (SMLMS) and the BioImage Informatics Conference (BII). This yielded a list of 150 challenges with 549 tasks.

DS2 - See Methods (Inclusion criteria for "Experiment: Sensitivity of challenge ranking"):
We faced several requirements with respect to the data acquisition: (1) The data should already have been published. As we started approaching challenge organizers in 2016, this meant that we were only able to include challenges up to the year 2015. On the other hand, (2) the data should reflect current practice (suggesting to use the latest data available), and (3) challenge organizers should still be approachable to allow for smooth data processing. Considering these conflicting requirements, we decided to include all segmentation challenges that were organized in 2015. Our analysis is based on a total of 124 tasks, hence reflecting 32% of all segmentation competitions organized to date.

Data exclusions

DS2:
In order to analyze rankings of biomedical challenges, challenge tasks were included according to following criteria (see Methods (Inclusion criteria for "Experiment: Sensitivity of challenge ranking")):
- Number of algorithms >= 3
- Number of test cases > 1 (for bootstrapping and cross-validation approaches)
- No explicit argumentation against the usage of Hausdorff Distance as metric

Replication

DS1 - See Methods (Experiment: Comprehensive reporting):
A tool for instantiating the challenge parameter list (Supplementary Table 2) introduced in the Methods section "Challenge parameter list" was used by some of the authors (engineers and a medical student) to formalize all challenges that met our inclusion criteria as follows: (1) Initially, each challenge was independently formalized by two different observers. (2) The formalization results were automatically compared. In ambiguous cases, when the observers could not agree on the instantiation of a parameter - a third observer was consulted, and a decision

	was made. When refinements to the parameter list were made, the process was repeated for missing values. Based on the formalized challenge data set, a descriptive statistical analysis was performed to characterize common practice related to challenge design and reporting.<br><br>The descriptive statistics have been implemented redundantly (Java and Python) for back-to-back comparison of generated values. Conflicts were resolved through discussion.<br><br>DS2:<br>In order to be able to verify the metric values provided by the challenge contact person, we asked them to provide additional measures, which were used as a simple "check sum" test for some metrics. For failing tests, we asked the contact persons for clarification and revision of the data.
Randomization	Randomization was not performed, because it was not relevant for this type of study.
Blinding	Blinding was not performed, because it was not relevant for this type of study.

Reporting for specific materials, systems and methods

Materials & experimental systems

n/a	Involved in the study	
☒	☐	Unique biological materials
☒	☐	Antibodies
☒	☐	Eukaryotic cell lines
☒	☐	Palaeontology
☒	☐	Animals and other organisms
☒	☐	Human research participants

Methods

n/a	Involved in the study	
☒	☐	ChIP-seq
☒	☐	Flow cytometry
☒	☐	MRI-based neuroimaging
